# Mutational insights and *in silico* characterization of *NEK* family kinases in OSCC patients from the Pakistani population

**DOI:** 10.3389/fbinf.2025.1750649

**Published:** 2026-02-04

**Authors:** Fouzia Nawab, Wafa Naeem, Sadia Fatima, Muhammad Uzair Khan, Aamir Mehmood, Sadia Nawab, Ishaq Khan, Haseena Nawaz, Hilal Ahmad, Ali Talha Khalil, Ishtiaq Ahmad Khan, Muhammad Irfan, Mohammed Alorini, Syed Ali Khurram, Asif Ali

**Affiliations:** 1 Institute of Basic Medical Sciences, Khyber Medical University, Peshawar, Pakistan; 2 Department of Bioinformatics and Biostatistics, State Key Laboratory of Microbial Metabolism and School of Life Sciences and Biotechnology, Shanghai Jiao Tong University, Shanghai, China; 3 School of Food Science and Engineering, South China University of Technology, Guangzhou, China; 4 Biofilm Laboratory, Research Centre for Life Science and Healthcare, Nottingham Ningbo China Beacons of Excellence Research and Innovation Institute (CBI), University of Nottingham Ningbo, Ningbo, China; 5 Department of Pathology, Lady Reading Hospital Medical Teaching Institution (LRH-MTI), Peshawar, Pakistan; 6 Jamil-ur-Rahman Center for Genome Research, Dr. Panjwani Center for Molecular Medicine and Drug Research, International Center for Chemical and Biological Sciences, University of Karachi, Karachi, Pakistan; 7 Department of Pathology, College of Medicine, Qassim University, Buraidah, Saudi Arabia; 8 Unit of Oral and Maxillofacial Pathology, School of Clinical Dentistry, University of Sheffield, Sheffield, United Kingdom; 9 Institute of Pathology and Diagnostic Medicine, Khyber Medical University, Peshawar, Pakistan; 10 School of Cancer Sciences, University of Glasgow, Glasgow, United Kingdom

**Keywords:** Oral squamous cell carcinoma (OSCC), whole exome sequencing (WES), NEK genes, biomarkers, *in silico* analysis

## Abstract

**Introduction:**

Oral squamous cell carcinoma (OSCC) is a prevalent malignancy characterized by aggressive behavior, poor prognosis, and limited therapeutic options. Mutations in the NIMA-related kinase (NEK) family are increasingly implicated in tumorigenesis across various cancers. However, their contributions to OSCC pathogenesis remain largely unexplored.

**Methods:**

Here, we employed whole-exome sequencing (WES) of formalin-fixed paraffin-embedded (FFPE) tissue blocks from 31 OSCC tumors and 9 adjacent paired normal samples derived from patients of Khyber Pakhtunkhwa (KP), Pakistan, to systematically profile *NEK* gene alterations. Subsequent *in-silico* analyses were performed to evaluate the structural and functional consequences of the identified mutations.

**Results:**

We identified 46 mutations overall (78.3% (36/46) somatic, 21.7% (10/46) germline), consisting of 82.6% (38/46) non-synonymous single-nucleotide variants (SNVs), 10.9% (5/46) frameshift deletions, 2.2% (1/26) non-frameshift deletions, and 4.3% (2/46) stop-gain mutations; notably, 10.9% (5/46) represented novel variants (not reported previously). NEK1 displayed the highest mutation frequency, followed by *NEK10, NEK5, NEK11, NEK2*, and *NEK3*. ISPRED-SEQ classified 37.0% (17/46) of mutations as residing at protein-protein interaction interfaces, indicating potential functional relevance, with several mutations including *NEK1p.D409Y, NEK1p.N643K, NEK9 p.H174Y, NEK10 p.R275C*, and *NEK10 p.E596K* predicted to be deleterious and destabilizing by multiple tools, occurring at conserved residues and altering structural stability via molecular dynamics simulations. Clinically, *NEK4* mutations were significantly associated with tumor site (P=0.02), *NEK9* with tobacco exposure (P=0.01), and *NEK10* with improved overall survival (P=0.01). Mutations including *NEK11p.E347V* (31/31), *NEK9p.R429H* (23/31), *NEK10p.L513S* (15/31), *NEK4p.P136A* (7/31), *NEK5p.K255Q* (6/31) and *NEK1 p.E650G* (5/31) were found to be recurring mutations and can be validated further in large-scale studies for biomarker applicability.

**Conclusion:**

Collectively, these findings suggest *NEK* mutations as candidate drivers of OSCC pathogenesis, underscoring their potential as prognostic biomarkers and therapeutic targets, particularly in tobacco-associated disease.

## Graphical Abstract

**Figure:**
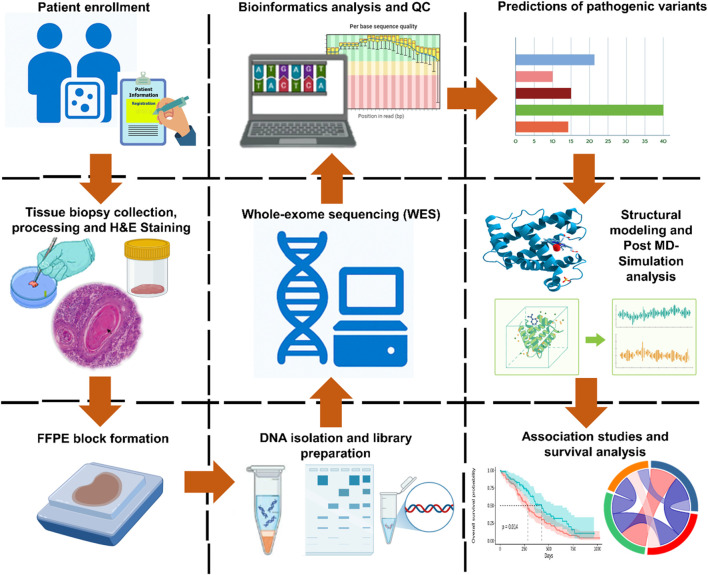

## Introduction

Oral squamous cell carcinoma (OSCC) is one of the most predominant malignant neoplasms of the oral cavity (Ferlay et al., 2021), constituting over 90% of oral cancer cases and ranking as the sixth most prevalent cancers globally. Arising from the epithelial lining of regions such as the buccal mucosa, tongue, mouth floor, and palate, OSCC is associated with substantial morbidity, mortality, and impaired quality of life (Rezazadeh et al., 2023). Based on the GLOBOCAN database (2022), oral cancer accounts for approximately ∼389,846 to 600,000 new cases and ∼188,438 deaths annually, with a significantly higher disease burden observed in South and Southeast Asia (Bray et al., 2024). In Pakistan, OSCC is the most frequently diagnosed cancer in men (12.3%) and the second most common cancer in women (5.3%), contributing to 8.6% of all cancer cases in 2022 (Ikram et al., 2023). In Western regions, including Europe and the United States, OSCC remains a persistent challenge, with over 130,000 and 34,000 new cases annually, respectively (Ghanem et al., 2024).

The etiological factors for OSCC are heterogeneous, with established risk factors such as tobacco use in both smoked and smokeless forms, alcohol consumption, snuff dipping, and betel quid chewing practices that are particularly common in South Asia (Gupta et al., 2016). Alarmingly, in 2022, an estimated 120,000 new oral cancer cases were linked to smokeless tobacco and areca nut use, particularly in South Central Asian regions and low- and middle-income countries (LMICs) (Warnakulasuriya et al., 2021). OSCC significantly influences the patients’ quality of life, leading to functional impairments, emotional distress, and social isolation. Despite developments in treatment techniques, the five-year survival rate for OSCC continues to fall below 50%, with women generally experiencing slightly better outcomes than men (Montero and Patel, 2015). This rising trend underscores the critical importance of developing effective methods for early diagnosis and improved therapeutic interventions. Surgery continues to be the leading treatment for oral cancer but often results in functional and esthetic impairments and requires long-term recovery and support (Dwivedi et al., 2020). Meanwhile, chemotherapy, radiotherapy, and emerging immunotherapeutic approaches are constrained by toxicity, intolerance, and limited efficacy (Raghavi and Anbarasu, 2024). Consequently, the identification of robust molecular biomarkers is critical for improving diagnostic accuracy and therapeutic efficacy.

Recent advances in molecular oncology have underscored the role of kinase gene mutations in the progression of OSCC (Su et al., 2017; Capra et al., 2006). Among these, the NIMA-related kinase (NEK) family has emerged as a key player in regulating a wide array of cellular processes, including apoptosis, DNA damage response, and cell-cycle regulation, particularly active in the S and G_2_/M phases, contributing to centrosome separation and mitosis^13^. Dysregulation and overexpression of *NEK* genes have been linked to poor prognosis, increased tumor aggressiveness, and therapeutic resistance in several cancers, suggesting that *NEK* genes may function as potential biomarkers and targets for cancer therapy (Cao et al., 2018).

Despite the growing recognition of *NEK*s in cancer biology, there remains a significant gap in understanding their mutational landscape in OSCC, especially within the Pakistani population. Building on our previous studies on the genetic landscape in oral cancer (Naeem et al., 2025; Nawab et al., 2025), this study aims to fill that gap by profiling mutations in selected *NEK* family genes using next-generation whole-exome sequencing (NG-WES) of OSCC samples of Khyber Pakhtunkhwa. Furthermore, by applying *in silico* analyses such as protein modeling and functional annotation, we seek to evaluate the biological impact of these mutations. This research could contribute to the identification of novel prognostic markers and therapeutic targets, ultimately supporting the advancement of precision oncology in OSCC.

## Materials and methods

### Patient selection

A total of 31 tumor tissue samples from OSCC patients and their 9 corresponding adjacent non-tumorous tissues (paired normal) were collected for the study.

### Inclusion criteria

Patients of both sexes and all age groups with a confirmed clinical and histopathological diagnosis of OSCC were included.

### Exclusion criteria

Patients presenting with recurrent tumors, a prior history of alternative treatments (such as radiotherapy/chemotherapy), or those diagnosed with malignancies other than OSCC were not enrolled in the study.

### Data collection and sample processing

Based on the inclusion criteria, tissue biopsies were derived from OSCC patients (following clinical and histological confirmation) recruited at two different hospitals of Peshawar, Pakistan (Hayatabad Medical Complex and Khyber College of Dentistry, Peshawar). Signed informed consent, along with patients’ history, demographic, and clinical details, was obtained prior to sample collection. The study was approved by the Ethical Committee of Khyber Medical University, Peshawar (Reference No. Dir/Ethics/KMU/2020/17; dated 29 January 2020), and all the procedures were conducted in accordance with the ethical standards of the Declaration of Helsinki. A certified pathologist identified the tumor and paired adjacent normal tissues. Tissue samples were fixed in formalin solution (10%) and processed into formalin-fixed, paraffin-embedded (FFPE) tissue blocks for subsequent analysis. Moreover, for histopathological evaluation, hematoxylin and eosin (H&E) staining was carried out on the FFPE block sections.

### Genomic DNA isolation and quality evaluation

Genomic DNA was isolated from FFPE tissue blocks with over 50% tumor cellularity using the Qiagen QIAamp DNA FFPE Tissue Kit (Cat. No. 56404) in accordance with the manufacturer’s protocol. To obtain sufficient DNA yield, a core measuring at least 2.5 mm diameter was taken from each tissue block. The quality of extracted DNA was examined through 2% agarose gel electrophoresis, while its concentration was determined using a high-sensitivity (HS) dsDNA Qubit kit on a Qubit fluorometer 2.0 (Thermo Fisher Scientific). DNA samples of suitable quality were stored at −20 °C till further analysis.

### Library preparation and whole-exome sequencing

For library preparation, high-quality genomic DNA (200 ng–300 ng) with an average fragment size >200 base pairs was selected, using Illumina’s DNA Prep with Exome 2.5 Kit, as per the manufacturer’s instructions. Genomic DNA was enzymatically fragmented, after which paired-end adapters were attached to the resulting fragments. Libraries were further amplified via limited-cycle PCR, followed by hybridization-based enrichment of exonic regions with coding exome (CEX) oligonucleotides. Post-capture libraries were amplified and purified with Agencourt AMPure magnetic beads. Library size distribution was measured using 2% agarose gel electrophoresis, and quantification was performed through the Qubit HS dsDNA assay (Thermo Fisher Scientific).

Enriched libraries were normalized to 20 (pmol) with HT1 buffer and subsequently diluted to 1.8 pmol for high-throughput paired-end sequencing (2 × 150 base pairs) on the Illumina NextSeq 500 platform using a 150-cycle flow cell. Final library dilution to 2 nM in 10 µL was used for cluster generation and sequencing, yielding a mean sequencing depth of ×100 across targeted exonic regions. The raw sequencing output was obtained in FASTQ format for downstream processing. Bedtools ‘coverage’ in combination with the GENCODE v47 gene annotation was used to compute per-exon coverage, resulting in an average depth of ×67.25 across exons.

### Analysis and annotation of sequence data

For sequencing data analysis, quality assessment of the raw reads was carried out using FASTQC to ensure data reliability (https://www.bioinformatics.babraham.ac.uk/projects/fastqc/) (Simon Andrews et al., 2010). FASTQC uses per-base/sequence quality estimates that are calculated using Phred scales, GC content calculations that are based on distribution differences from an expected distribution, and estimates of sequence duplication using k-mer profiling. Good quality sequencing data in the FASTQ format were aligned to the hg38 UCSC reference genome using the BWA (Burrows–Wheeler Aligner) tool. BWA-MEM proceeds with a seed-and-extend algorithm with seeds that are maximal exact matches, referred to as MEMs. Alignment scoring for BWA-MEM uses affine gap penalties with dynamic programming, while the probability-based model of uncertain alignments estimates mapping quality (Li and Durbin, 2009; Li, 2013). After alignment, the resulting BAM files were sorted, and PCR duplicates were deleted with the help of the Picard tool 1.109. Base quality score recalibration and variant calling were performed following the standard Genome Analysis Toolkit (GATK) pipeline (McKenna et al., 2010; DePristo et al., 2011; der Auwera et al., 2013). GATK HaplotypeCaller ascertains candidate haplotypes by *de novo* assembly of the graph of reads and infers a pair HMM-based probability of a given haplotype being true, given a set of reads. Genotype calling then follows a Bayesian scheme that ascertains genotype posteriors from a set of allele probabilities, assuming Hardy–Weinberg equilibrium.

To ensure the robustness and high-quality variants detection, only variants with a quality score (QUAL) >30, read depth (DP) <20, genotyping quality (GQ) above 20, and minor allele frequency (MAF) <0.01 in the gnomAD v4.1 were retained. Furthermore, variant annotation was performed with ANNOVAR to generate a detailed CSV file containing all the functional and genomic information. The read counts were normalized using factors such as TPM, RPKM, or depth-scaled approaches, which rely on linear scaling equations that incorporate reads per gene size and total library size to facilitate comparable results among different samples. Subsequent data filtration and analysis were carried out using the R program.

### Bioinformatics analysis

All the variants’ pathogenicity was assessed using different *in silico* prediction tools: SIFT (Sorting Intolerant From Tolerant), PolyPhen-2 (polymorphism phenotyping version 2), MutationTaster, MutationAssessor, PROVEAN (Protein Variation Effect Analyzer), and FATHMM (Functional Analysis Through Hidden Markov Models). ISPRED-SEQ (https://ispredws.biocomp.unibo.it/sequence/) was used for predicting interaction sites (ISs) in protein sequences. SAAFEC-SEQ (http://compbio.clemson.edu/lab/) was used to predict the impact of mutations on the stability of proteins. The ConSurf web server (https://consurf.tau.ac il/) was used to examine the evolutionary conservation of the altered residues and predict their functional and structural characteristics (including whether they were buried or exposed within the protein). Furthermore, a gene-wise comparison of *NEK* family mutation frequencies was performed between the study cohort and TCGA-HNSCC dataset (n = 515) using mutation data retrieved from cBioPortal. Frequencies were calculated as the proportion of samples with at least one mutation per *NEK* gene and summarized graphically.

### Mutation mapping and structural modeling

Lollipop plots were generated using the “Maftools” package in RStudio to visualize the distribution and location of different mutations across *NEK* genes (Mayakonda et al., 2018). To predict the potential impact of the mutated residue on protein structure and functions, another online web-based tool, namely, “HOPE” (https://www3.cmbi.umcn.nl/hope), was used. For structural modeling, mutant proteins (with altered residues at the interaction site) were modeled using “SWISS-MODEL” (https://swissmodel.expasy.org/). The wild and mutant proteins were then superimposed and visualized using PyMOL. Moreover, STRING and GeneMANIA platforms were utilized to assess protein–protein interaction networks and functional associations.

### Association with clinicopathological parameters and overall survival

The mutational spectrum of *NEK* genes was analyzed in relation to clinicopathological (age, gender, tumor grade, and tumor site) and sociodemographic factors (naswar use, smoking status, family history, and dental history) using OriginPro 2025 software. Chi-squared/Fisher’s exact test was used to determine the significant association of genes with determinants. Survival analysis was carried out using SPSS software, and comparisons between groups were conducted using the log-rank test to evaluate the impact of specific gene mutations on patient prognosis.

#### Molecular dynamics simulation (MDS)

**FIGURE 1:**
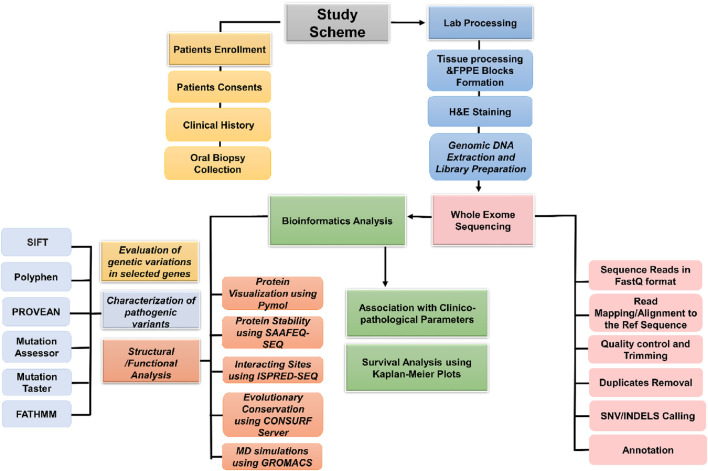
Study scheme.

GROMACS 5.1 was used for the simulations of selected interacting site proteins to evaluate their structural integrity and dynamic properties. Initial 3D protein models were generated using SWISS-MODEL, and the topology files were prepared with the OPLS-AA/L all-atom force field. Proteins were solvated in a cubic box using the SPC216 water model and neutralized with counter-ions to eliminate net charges. The system underwent energy minimization to remove steric clashes, followed by equilibration in two phases: NVT, to stabilize temperature, and NPT, to stabilize pressure and density under physiological conditions. A 100-ns production run was then conducted for both wild and mutant type (WT:MT) proteins. Post-simulation analyses included root mean square deviation (RMSD), root mean square fluctuation (RMSF), and radius of gyration (Rg), and thermodynamic parameters (density, temperature, pressure, and potential energy) were monitored throughout the simulation (Mehmood et al., 2023). Results were generated using OriginPro version 2025, and structural validation was performed via Ramachandran plots generated through the PROCHECK server. A schematic diagram of the study is shown in Figure 1.

## Results

### Demographic characteristics

**FIGURE 2:**
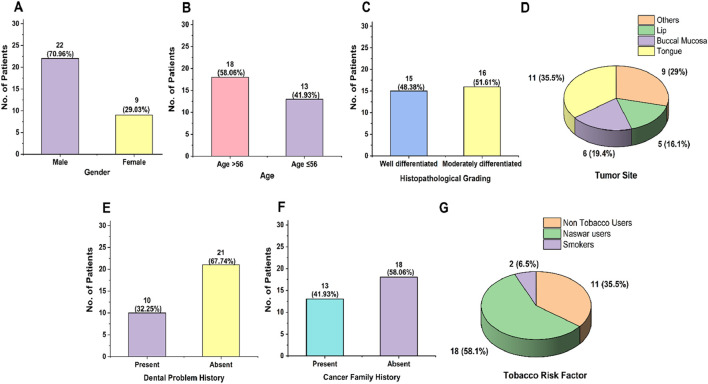
Overview of demographic and clinicopathological characteristics of the oral cancer patient cohort: **(A)** gender-wise distribution, **(B)** age-wise distribution, **(C)** histopathological grading, **(D)** tumor site distribution, **(E)** history of dental problems, **(F)** family history of cancer, and **(G)** distribution of tobacco-associated risk factors.

Figures 2A–G reveal the general demographic characteristics of the study. A total of 31 patients fulfilling the inclusion criteria participated in the study, comprising 22 men and 9 women, with men representing a high prevalence of OSCC cases (70.96%; 22/31). The majority of the participants were aged above 56 years (58.06%; 18/31). Histopathological grading revealed that 48.38% (15/31) were classified as well-differentiated and 51.61% (16/31) were classified as moderately differentiated. Anatomically, the tumors were distributed across several sites, including the tongue (35.48% cases; 11/31), lip (16.12% cases; 5/31), buccal mucosa (19.35% cases; 6/31), and other areas, including the oral cavity, mandible, palate, and mouth floor (29.03% cases; 9/31). Regarding sociodemographic factors, concerning tobacco use, 58.06% (18/31) used naswar, 6.45% (2/31) were smokers, and 35.48% (11/31) were non-tobacco users. A family history of cancer was reported in 41.93% (13/31) of patients. Additionally, 32.25% (10/31) had a history of dental issues such as infection or swelling.

### Mutational spectrum

**FIGURE 3:**
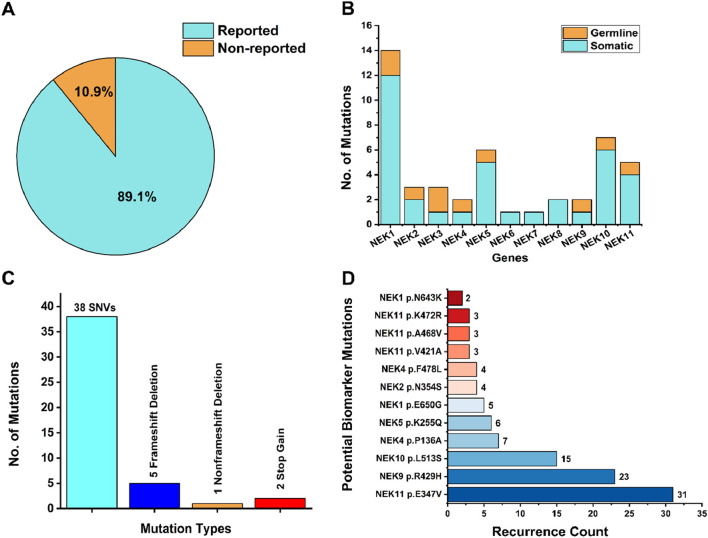
Mutational profile of NEK genes. **(A)** Percentage distribution of novel and previously reported mutations. **(B)** Distribution of somatic and germline mutations. **(C)** Mutation types across selected genes based on the mutation rate. **(D)** Recurrent mutations with biomarker potential significance.

The WES results of the selected genes were analyzed using mutation databases, including COSMIC and dbSNP. The overall mutational spectrum of the *NEK* genes is summarized in Supplementary Table S1. Gene variants identified exclusively in tumor samples were considered somatic mutations, whereas variants detected in both tumor and paired normal tissues were categorized as germline mutations. In total, 46 mutations were identified in all 11 *NEK* genes. Among the identified mutations, 38 were reported as non-synonymous single-nucleotide variants (SNVs) (38/46; 82.6%), five were frameshift deletions (5/46; 10.86%), 1 was identified as non-frameshift deletion (1/46; 2.17%), and two were identified as stop-gain mutations (2/46; 4.34%) (Figure 3C). Moreover, of the total mutations in all the 11 *NEK* genes, 36 (36/46; 78.26%) were classified as somatic mutations and 10 (10/46; 21.74%) were identified as germline mutations based on the comparison between 31 tumor and 9 adjacent paired normal tissues (Figure 3B). Meanwhile, 5/46 (10.9%) of the mutations were not reported previously (Figure 3A) and are considered novel mutations. These novel mutations included *NEK1* ^p.L270Vfs*2^, *NEK1* ^p.K347Efs*13^, *NEK1* ^p.E624Rfs*19^, *NEK1* ^p.N953Kfs*48^, and *NEK5* ^*p.D742del*^.

Gene-wise distribution of somatic mutations on all the 11 *NEK* genes were *NEK1*, 33.33% (12/36); *NEK2*, 5.55% (2/36); *NEK3*, 2.77% (1/36); *NEK4*, 2.77% (1/36); *NEK5*, 13.80% (5/36); *NEK6*, 2.77% (1/36); *NEK7*, 2.77% (1/36); *NEK8*, 5.55% (2/36); *NEK9*, 2.77% (1/36); *NEK10* 16.66% (6/36); and *NEK11*, 11.11% (4/36). However, mutations in *NEK11*, i.e., *NEK11* ^*p.E347V*^, were found in 100% of oral cancer patients (31/31), whereas *NEK9* ^*p.R429H*^ (74.19%; 23/31) and *NEK10* ^*p.L513S*^ (48.38%; 15/31) were detected in the majority of the cases, highlighting their potential as biomarkers within the local population. Similarly, *NEK4* ^*p.P136A*^ (22.58%; 7/31), *NEK5* ^*p.K255Q*^ (19.35%; 6/31), and *NEK1* ^*p.E650G*^ (16.12%; 5/31) were found to be recurring mutations and could be further investigated in larger cohorts to explore their potential as biomarkers (Figure 3D).

**FIGURE 4:**
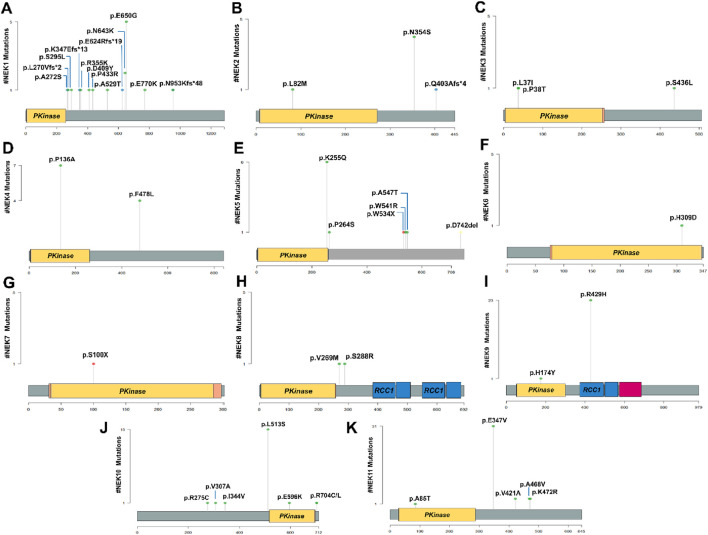
Distribution patterns of different mutations in *NEK* genes illustrated by lollipop plots. **(A)** *NEK1*, **(B)** *NEK2*, **(C)** *NEK3*, **(D)** *NEK4*, **(E)** *NEK5*, **(F)** *NEK6*, **(G)** *NEK7*, **(H)** *NEK8*, **(I)** *NEK9*, **(J)** *NEK10*, and **(K)** *NEK11*. The plots were created using “Maftools” package in RStudio.

Figures 4A–K present lollipop plots depicting the identified mutations and their respective locations on each gene.

**FIGURE 5:**
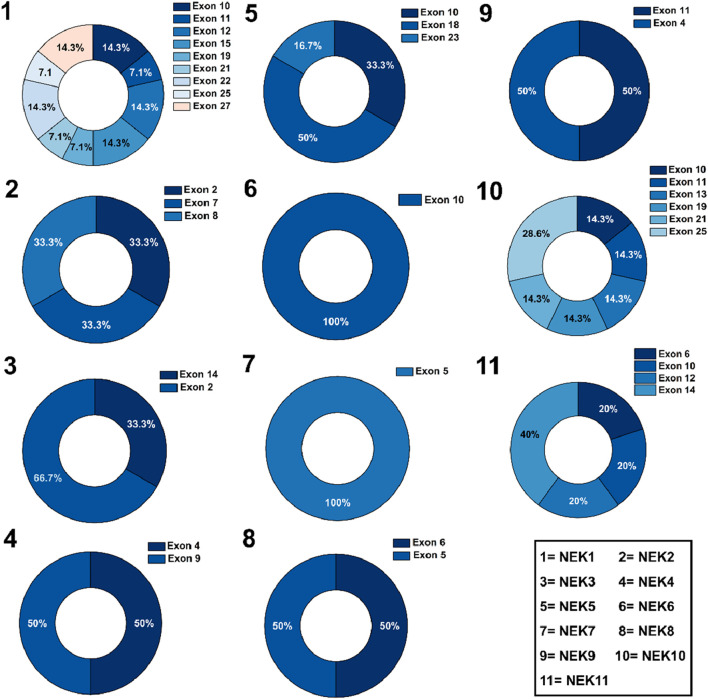
Exon-wise distribution of different mutations in *NEK* genes.

Figure 5 shows the exon-wise distribution of mutations in all the 11 *NEK* genes, with the highest mutation frequencies revealed in exons 2, 10, 12, 14, 15, 18, 22, 23, 25, and 27, respectively.

### Comparison of *NEK* gene mutation frequencies with TCGA-HNSCC

**FIGURE 6:**
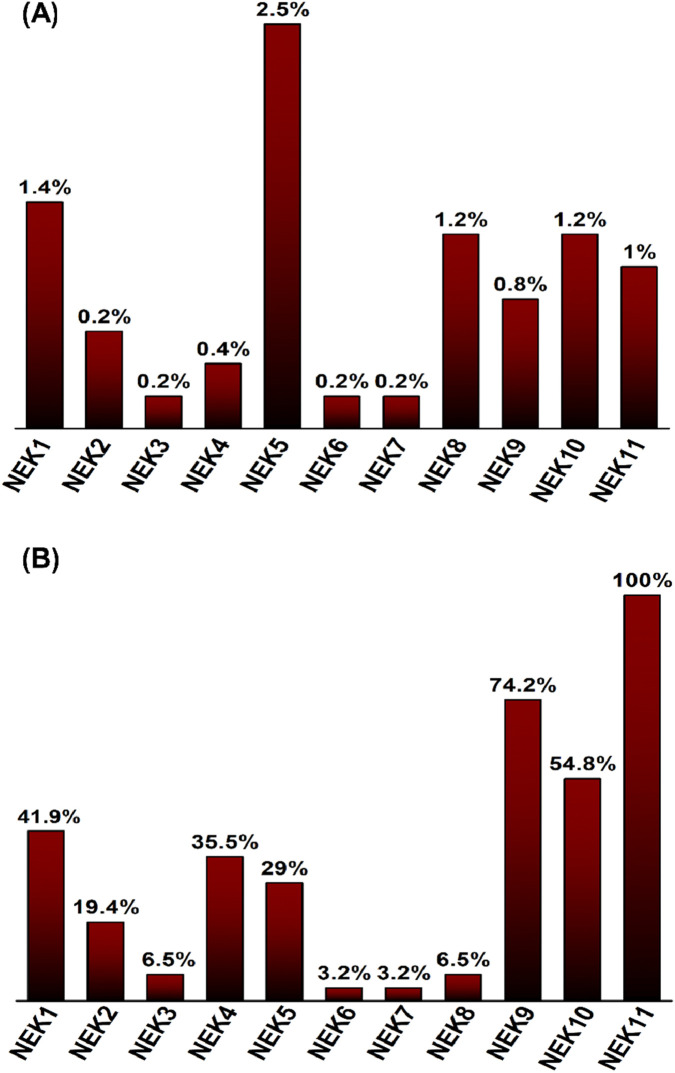
Comparative analysis of *NEK* gene mutation frequencies. **(A)** Mutation frequencies in TCGA-HNSCC dataset (n = 515), with NEK5 showing the highest rate (2.5%). **(B)** Mutation frequencies in the Pakistani OSCC cohort (n = 31), with *NEK11* exhibiting the highest rate (100%).

In the study cohort, among the mutated genes, *NEK11* mutations were most recurrent, being present in all samples (31/31; 100%), followed by *NEK9* in 23/31 (74.2%), *NEK10* in 17/31 (54.8%), *NEK1* in 13/31 (41.9%), and *NEK4* in 11/31 patients (35.5%). By contrast, mutation frequencies in TCGA-HNSCC were consistently low, with *NEK5* exhibiting the highest rate (13/515; 2.5%) and most *NEK* genes showing rates below 1%. These comparative gene-specific frequencies are summarized in Supplementary Table S6 and are depicted in Figure 6.

### Pathogenicity predictions of SNVs

**FIGURE 7:**
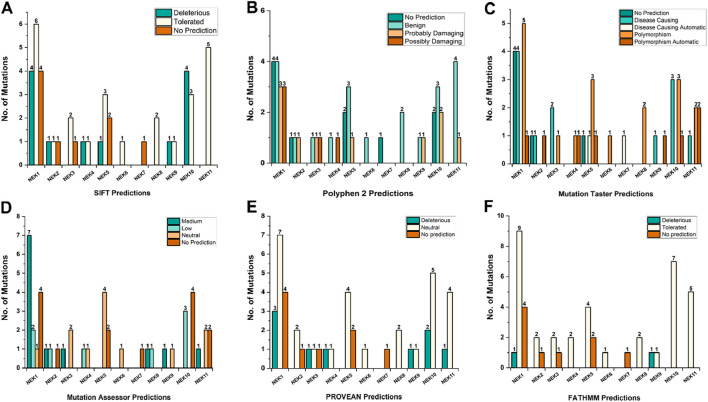
Pathogenicity predictions of *NEK* gene mutations based on SIFT **(A)**, PolyPhen-2 **(B)**, MutationTaster **(C)**, MutationAssessor **(D)**, PROVEAN **(E)**, and FATHMM **(F)** tools.

The *NEK* gene mutations were evaluated for pathogenicity in six different *in silico* prediction tools, namely, SIFT, PolyPhen-2, MutationTaster, MutationAssessor, PROVEAN, and FATHMM, based on criteria such as evolutionary conservation, protein structure, and biochemical properties (Liu et al., 2016). The predicted pathogenicity results are summarized in Figures 7A–F.

According to SIFT, of the 46 total mutations, 12/46 (26.08%) predictions were considered deleterious in nature. However, 9/46 (19.56%) mutations yielded no results. Gene-wise SIFT analysis showed the highest deleterious prediction rate in the *NEK10* gene, i.e., 4/7 (57.14%), followed by *NEK4*, 1/2 (50%); *NEK9*, 1/2 (50%); *NEK2*, 1/3 (33.3%); *NEK1*, 4/14 (28.57%); and *NEK5*, 1/6 (16.66). However, for *NEK3*, *NEK6*, *NEK7*, *NEK8*, and *NEK11*, none of the mutations were predicted as deleterious (Figure 7A). Similarly, PolyPhen-2 classified 3/14 (21.42%; *NEK1*), 1/3 (33.33%; *NEK2*), 1/3 (33.33%; *NEK3*), 1/6 (16.66%; *NEK5*), 1/2 (50%; *NEK9*), 1/7 (14.28%; *NEK10*), and 1/5 (20%; *NEK11*) mutations as probably damaging (Figure 7B). The MutationTaster database revealed 12/46 (26.08%) mutations as disease-causing across six genes (*NEK1*, *NEK2*, *NEK3*, *NEK9*, *NEK10*, and *NEK11)* (Figure 7C). Similarly, based on the MutationAssessor results, a subset of variants were classified under the medium- and low-impact categories; for genes including *NEK1*, *NEK2*, *NEK3*, *NEK8*, *NEK9*, and *NEK11*, 7/14 (50%), 1/3 (33.33%), 1/3 (33.33%), 1/2 (50%), 1/2 (50%), and 1/5 (20%) of the mutations, respectively, were categorized as having medium impact (Figure 7D). PROVEAN predicted 9/46 mutations (19.56%) as deleterious. In comparison, FATHMM predicted 2/46 mutations (4.37%) as deleterious in *NEK1* (*NEK1* ^*p.N643K*^) and *NEK9* (*NEK9* ^*p.R429H*^) genes, respectively (Figures 7E,F).

Another prediction tool, SAAFEC-SEQ, was utilized to evaluate the influence of SNVs on protein stability. All the identified SNVs were predicted to exert a destabilizing effect on the protein structure, as indicated in Supplementary Table S2. Additionally, ISPRED-SEQ identified 17 of 46 mutations (36.95%) as interaction-site mutations across all NEK genes, suggesting potential functional relevance due to their location at protein–protein interaction interfaces. The remaining 29 mutations (63.04%) were classified as non-interacting site mutations, exhibiting probability scores below the 0.5 threshold (Supplementary Table S3).

### Evolutionary conservation predictions

**FIGURE 8:**
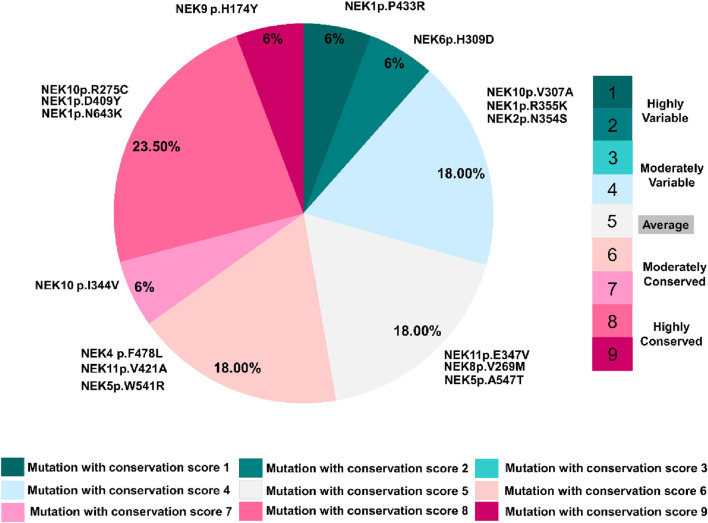
Conservation profile of mutated amino acid residues in *NEK*s, as predicted by ConSurf analysis.

The ConSurf tool was used to determine the evolutionary conservation of the altered residues. Figure 8 and Supplementary Table S4 show the conservation scores, which represent each residue’s structural and functional relevance. Results revealed that the interaction-site mutations in *NEK1* (*NEK1* ^*p.D409Y*^ and *NEK1* ^*p.N643K*^), *NEK9* (*NEK9* ^*p.H174Y*^), and *NEK10* (*NEK10* ^*p.R275C*^) are located in highly conserved areas, with scores of 8 and 9. *NEK1* ^*p.D409Y*^ and *NEK10* ^*p.R275C*^ were exposed and functional residues, respectively, whereas *NEK9* ^*p.H174Y*^ was projected to be a structural residue with a buried nature. Furthermore, overall, 24% of mutations (score 6–7) were moderately conserved with exposed/buried residue positions, suggesting a potential structural and functional impact. In contrast, 30% of mutations (scores 1, 2, and 4) were located at variable sites. However, 18% of the residues exhibited an average conservation score of 5, with either an exposed or a buried nature.

## MD simulations

For molecular dynamic simulations, five mutations (*NEK1* ^*p.D409Y*^, *NEK1* ^*p.N643K*^, *NEK9* ^*p.H174Y*^, *NEK10* ^*p.R275C*^, and *NEK10* ^*p.E596K*^) were prioritized based on their predicted pathogenicity, as identified as deleterious by 5–4 different bioinformatics tools. All these mutations were located at critical interaction sites within the protein structure, suggesting a likely impact on protein stability and function. All the mutant types of the proteins maintained consistently higher Rg values than their WT counterparts throughout the 100-ns simulation, indicating potential structural destabilization (Supplementary Figure S1C–S5C). The average radius of gyration for all the mutations is shown in Supplementary Figure S6. Among the analyzed mutations, *NEK1* ^*p.D409Y*^ exhibited an average radius of gyration (Rg) of 5.62 nm for the mutant protein and 4.45 nm for the WT. Similarly, for *NEK9* (*NEK9* ^*p.H174Y*^) and *NEK10* (*NEK10* ^*p.R275C*^ and *NEK10* ^*p.E596K*^) IS mutations, the average Rg values for the mutant protein were higher than those of the WT (Supplementary Figure S6). RMSD analyses revealed substantial structural deviations in mutant proteins compared to those in WT. All the *NEK* gene mutants exhibit consistently higher backbone deviations compared to the WT throughout the 100-ns simulation. *NEK1* mutations, i.e., *NEK1* ^*p.D409Y*^ and *NEK1* ^*p.N643K*^, showed a substantial deviation, particularly after 20 and 40 ns, as indicated in Supplementary Figures S4A, S5A. *NEK9* ^*p.H174Y*^ exhibited major deviations after 10 ns and minor deviations after 50 ns (Supplementary Figure S3A). Similarly, *NEK10* mutants (*NEK10* ^*p.R275C*^ and *NEK10* ^*p.E596K*^) exhibited greater structural deviations than WT, indicating reduced conformational stability (Supplementary Figures S1A, S2A).

**FIGURE 9:**
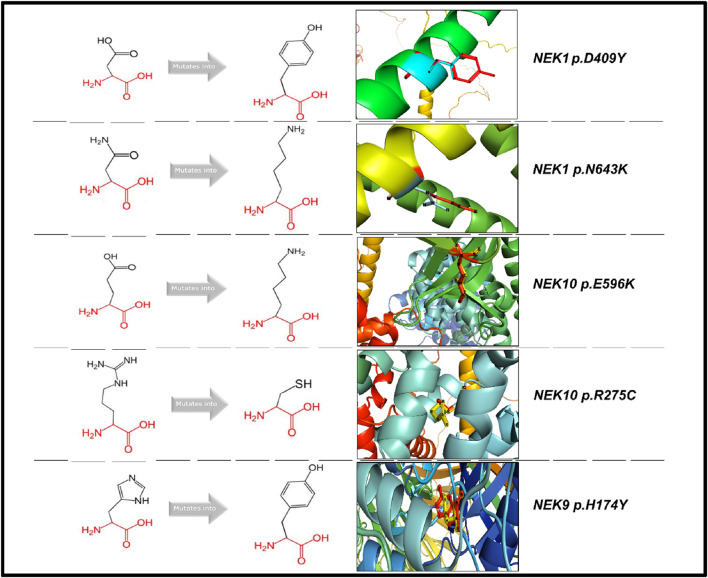
Visualization and superimposition of interacting-site mutations in PyMOL with HOPE-predicted structural effects.

RMSF profiles further supported these findings. All the mutants exhibited increased flexibility around the mutated residues compared to WT proteins, suggesting increased local flexibility and potential functional disruption (Supplementary Figures S1B–S5B). In addition, other energy parameters (i.e., density, temperature, and pressure) also showed noticeable fluctuations in mutant proteins, supporting the hypothesis of compromised structural stability due to IS mutations. Ramachandran plots were also generated, indicating minor changes in residue conformational distributions (Supplementary Figures S1–S5H,I). All these interacting-site mutations were superimposed and visualized using PyMOL. HOPE analysis further predicted that these substitutions may induce structural destabilization and functional alterations in the proteins, as illustrated in Figure 9.

### Gene-wise association with histopathological grading and sociodemographic parameters

**FIGURE 10:**
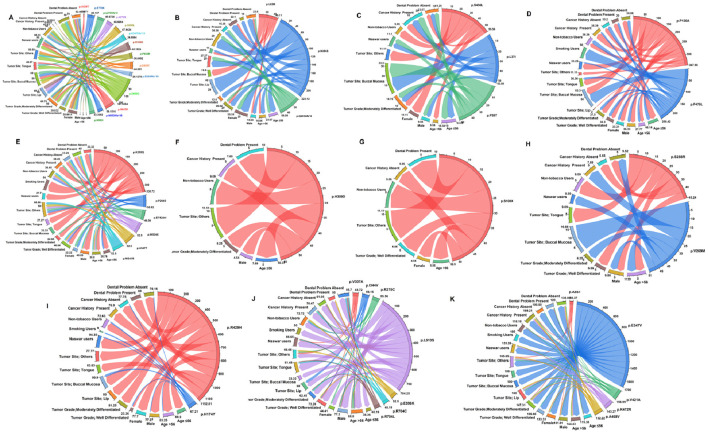
Association of *NEK* gene mutations with histopathological and sociodemographic characteristics: **(A)** *NEK1*, **(B)** *NEK2*, **(C)** *NEK3*, **(D)** *NEK4*, **(E)** *NEK5*, **(F)** *NEK6*, **(G)** *NEK7*, **(H)** *NEK8*, **(I)** *NEK9*, **(J)** *NEK10*, and **(K)** *NEK11*.

**FIGURE 11:**
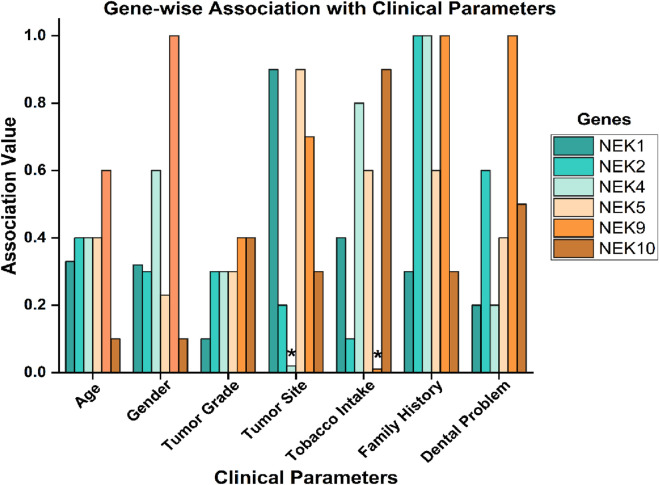
Association of *NEK* gene mutations with histopathological and sociodemographic characteristics; *, significant (p-value < 0.05)*.*

To assess the clinical relevance of *NEK* mutations in oral cancer, we examined their association with sociodemographic and histopathological parameters (Figures 10A–K). Statistically significant associations were identified for *NEK4* and *NEK9*. *NEK4* mutations were significantly linked to the tumor site (p = 0.02), suggesting a potential role in site-specific tumorigenesis. *NEK9* mutations showed a strong association with tobacco intake (p = 0.01), indicating its possible involvement in tobacco-related oral carcinogenesis. In contrast, no significant associations were observed for *NEK1*, *NEK2*, *NEK5*, or *NEK10* (Figure 11; Supplementary Table S5A,B).

### Association with overall survival

**FIGURE 12:**
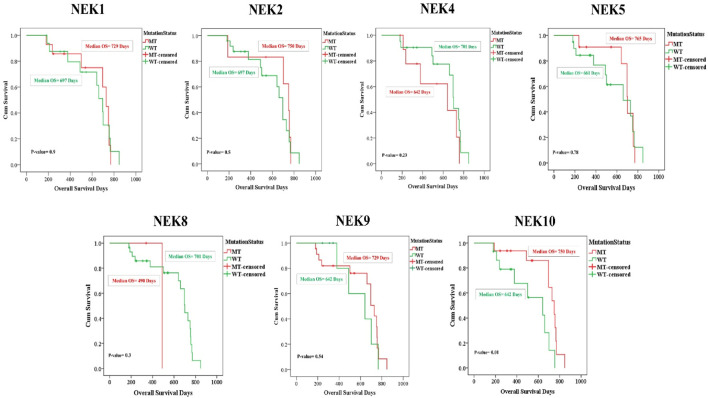
Gene-wise association of NEK genes with overall survival (p-value < 0.05 is considered significant).

Kaplan–Meier analysis was performed using SPSS to analyze the prognostic relevance of mutations in *NEK* genes in OSCC patients (Figure 12). A statistically significant difference in improved overall survival was observed between patients harboring *NEK10* mutations and those with WT *NEK10* (p < 0.05), indicating a potential role of *NEK10* mutations in patient prognosis. Similarly, mutations in *NEK1*, *NEK2*, *NEK5*, and *NEK9* genes did not exhibit statistical significance (with p-values of 0.9, 0.5, 0.7, and 0.5, respectively). However, patients carrying mutations in these genes tended to show better overall survival compared to their WT counterparts. In contrast, a non-significant decrease in overall survival was seen in patients having mutations in the *NEK4* and *NEK8* genes compared to their WT counterparts.

## Discussion

Identifying reliable genetic biomarkers in oral squamous cell carcinoma remains a central challenge. Despite advances in cancer genomics, the molecular architecture of OSCC has not been fully elucidated, particularly regarding the contribution of underexplored kinases such as the NIMA-related kinase (*NEK*) family. *NEK*s are a family of 11 serine/threonine kinases (*NEK1–NEK11*) emerging as critical regulators of various mitotic processes (cell-cycle regulation, apoptosis, centrosome function, spindle assembly, and the G_2_/M transition processes) that are frequently dysregulated in cancer (Nguyen et al., 2023). By maintaining genomic stability and cellular homeostasis, these kinases play an essential role in regulating normal cellular functions. In this study, we present the first comprehensive mutational profiling of all 11 *NEK*s in OSCC patients from Khyber Pakhtunkhwa (KPK), revealing novel insights into their demographic associations, mutation spectra, and prognostic relevance/significance. Furthermore, we carried out *in silico* analysis to determine the pathogenic significance and potential effects of the variants on protein function using multiple bioinformatics tools.

Using WES of 31 tumor tissues, we identified multiple somatic and germline variants (36/46; 78.26% and 10/46; 21.74%) in *NEK* genes, several of which are predicted to be pathogenic (*NEK1*, *NEK2*, *NEK5*, *NEK9*, *NEK10*, and *NEK11* mutations are deleterious) based on consensus outputs from SIFT, PolyPhen-2, and other pathogenicity tools, as reported above. Among these genes, *NEK1* (14/46; 30.43%), *NEK5* (6/46; 13.04%), *NEK10* (7/46; 15.21%), and *NEK11* (5/11; 10.86%) showed the highest mutation burden. These findings highlight the potential oncogenic relevance of *NEK*s in OSCC. Our results are in line with their previously reported oncogenic roles in other cancer types, including ovarian, lung, brain, breast, and colorectal cancers (Fry et al., 2012; Chen et al., 2025; Tsunoda et al., 2009). Previous literature reports that *NEK10* has been identified as one of the important kinases likely harboring driver mutations, based on cancer whole-genome sequencing datasets, with 13 cataloged missense variants across multiple tumor types (Greenman et al., 2007). Our study also supports this finding, revealing a high frequency of *NEK10* mutations in oral squamous cell carcinoma patients from Pakistan. The non-synonymous SNVs (*NEK10* ^*p.R275C*^ and *NEK10* ^*p.E596K*^) were reported as the most deleterious mutations in *NEK10*, which occur at the interacting site and have a destabilizing impact on protein structure. Similarly, *NEK1* mutations have been documented in diverse cancers such as ovarian, colorectal, lung, and skin tumors, where *NEK1* dysfunction is linked to chromosomal instability and defective DNA damage checkpoint phenotypes that promote malignant transformation in both cellular and animal models (Chen et al., 2011). *NEK11* has also been frequently implicated in somatic alterations across various cancers. *NEK11* exhibits both oncogenic and tumor-suppressive functions across different cancer types (Li H. et al., 2025). Exome sequencing studies in melanoma and colorectal cancers have identified several mutations in *NEK11*. Functional analyses further reveal that loss of *NEK11* disrupts the G_2_/M cell-cycle checkpoint in response to DNA damage, leading to heightened genomic instability and reduced apoptosis, thereby potentially promoting tumor progression (Sabir et al., 2015). However, *NEK11* ^*p.E347V*^ was one of the most common mutations in our population, which was frequently reported in all 31 OSCC patients.

*NEK*s play key roles in regulating mitotic progression, centrosome dynamics, spindle formation, and genomic stability. Dysregulation of these processes is a hallmark of cancer, and alterations in *NEK* genes have been linked to abnormal proliferation, chromosomal instability, and enhanced metastatic behavior in multiple malignancies. Mutations identified in this study, particularly those predicted as damaging, may impair catalytic activity or substrate binding, disrupting cell-cycle checkpoints and mitotic control. These changes can also interact with key OSCC pathways such as PI3K/AKT, MAPK, and DNA damage response, underscoring the role of NEKs in tumorigenesis and as potential biomarkers (Fry et al., 2012; Moniz et al., 2011; Xie et al., 2020). The inclusion of TCGA-HNSCC data places our findings within a broader genomic context and supports the relevance of the identified genes in head and neck cancers. Although mutation frequencies were comparatively higher in our cohort, TCGA analysis confirms that these genes are not unique to our dataset but are recurrently altered in larger populations. Given the exploratory nature of this study and the modest sample size, these observations should be interpreted cautiously and warrant validation in larger, independent cohorts.

**FIGURE 13:**
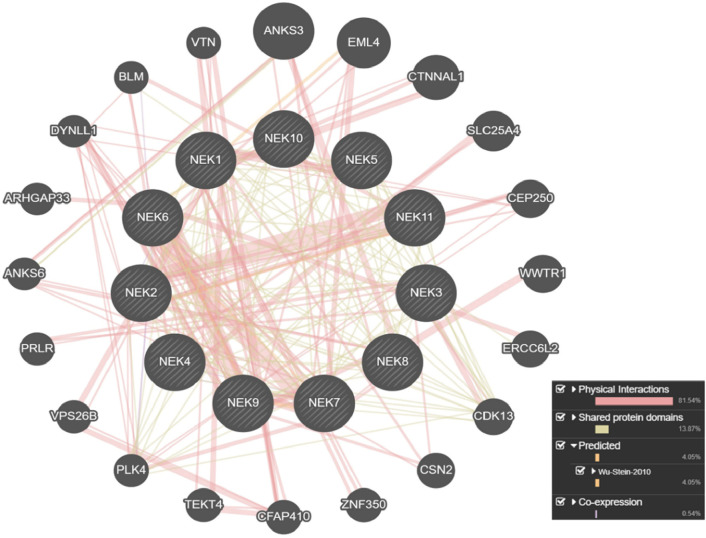
Physical interaction network of NEK genes as predicted by the GeneMANIA server.

Furthermore, the convergence of computational predictions strengthens the conclusion that these mutations are likely impactful in OSCC pathogenesis. All these genes have destabilized proteins. Nearly 41% (19/46) coincide with protein–protein interaction sites, and key mutations in *NEK1* (*NEK1* ^*p.D409Y*^ and *NEK1* ^*p.N643K*^), *NEK9* (*NEK9* ^*p.H174Y*^), and *NEK10* (*NEK10* ^*p.R275C*^) occur in highly conserved, functionally critical regions, with scores of 8 and 9. Using the STRING server and GeneMANIA, we analyzed the interaction networks for these genes (Figure 13; Supplementary Figure S7). The resulting interactome pathways reveal that these proteins participate in multiple biological processes associated with genome integrity, and any mutation in them may disrupt these interactions, ultimately contributing to tumor progression. These *in silico* findings extend observations of *NEK*s influencing mitotic regulation, DNA repair, and signal transduction pathways (Zhu et al., 2024).

Recent advances in computational and deep learning methods have enhanced mutation detection, functional impact prediction, and biomarker prioritization in cancer genomics. For instance, heterogeneous information network learning models with neighborhood-level structural representation predict biologically relevant interactions, such as lncRNA–miRNA networks (Zhao et al., 2024), whereas geometric deep learning frameworks enable drug repositioning over heterogeneous networks (Zhao et al., 2022). Similarly, sequence-based deep learning models accurately predict functional sites, including HIV-1 protease cleavage sites (Li D. et al., 2025), and structure-based and molecular modeling advances, such as fine-tuned MM/PBSA (GBSA)-based methods and multi-perspective 3D molecular representations, improve variant interpretation and clinically relevant biomarker identification (Zhang et al., 2025; Zhao et al., 2025).

Although the present study employed traditional *in silico* tools to assess mutation impact and protein–protein interactions, recent advancements in machine learning and deep learning are revolutionizing the interpretation of cancer-associated mutations and network-level analysis. Ensemble approaches, such as rotation forest models, enhance accuracy in disease–gene association predictions by integrating multi-feature similarity profiles. Applying analogous ensemble or deep learning strategies to *NEK* gene mutations could provide enhanced insights into mutation pathogenicity, protein–protein interaction disruptions, and network-level effects that are not fully captured by conventional methods (Guo et al., 2019).

Furthermore, current trends in computational PPI prediction, propelled by modern machine learning and deep learning frameworks, reflect a transition from traditional sequence-based tools such as ISPRED-SEQ. Modern approaches integrate structural and evolutionary protein features to more accurately assess mutation-induced interaction changes. Methods including AlphaFold Multimer, graph neural network (GNN)-based PPI predictors, and transformer-based protein language models have shown improved performance in identifying interaction interfaces and evaluating the functional consequences of variants (Wang Y. et al., 2020; Wang et al., 2019). These transformer-based models learn contextual relationships across protein sequences, enabling detection of long-range dependencies and subtle sequence–structure effects that are often missed by conventional tools. Incorporating such approaches into future analyses of *NEK* gene mutations in oral cancer may enhance the identification of critical PPI disruptions, support prioritization of variants for experimental validation, and strengthen the interpretability of *in silico* findings.

Additionally, recent deep learning approaches have significantly enhanced drug–target interaction (DTI) prediction and hold relevance for future *NEK* druggability assessment. LSTM-based neural networks and transformer models capture complex protein–ligand interactions, predicting potential small-molecule binders more accurately than traditional docking or similarity-based tools. These methods could prioritize selective *NEK* inhibitors and guide therapeutic development, as supported by recent studies on LSTM-based DTI frameworks that underscore the benefits of integrating such models into *NEK*-focused drug discovery (Wang Y.-B. et al., 2020).

The somatic mutational profile of cancers is strongly influenced by inherited genetic predispositions and the distribution of allele frequencies within specific populations. In regions characterized by rich ethnic diversity, such as South Asia, these factors become particularly significant. Our cohort from KPK reflects a genetically distinct ethnic community with unique hereditary backgrounds and characteristic lifestyle exposures, including the widespread use of naswar, i.e., smokeless tobacco, smoking, and betel nut consumption (IARC Working Group on the Evaluation of Carcinogenic Risks to Humans, World Health Organization and International Agency for Research on Cancer, 2004; Ferlay et al., 2020; India Project Team of the International Cancer Genome Consortium, 2013). These carcinogenic factors are known to cause DNA damage and may potentially alter the pattern and frequency of mutations in *NEK* genes (Sharan et al., 2012). We observed a higher incidence of OSCC in men (71%) and individuals older than 56 years (58%), which is consistent with global trends of OSCC prevalence in men and older age groups. Notably, naswar use emerged as the primary risk factor (58%), while a significant subset of patients also presented with dental issues (32%). These findings align with prior studies linking smokeless tobacco and poor oral hygiene to OSCC risk, reinforcing the role of these exposures in regional populations (Chen et al., 2025). Furthermore, *NEK4* and *NEK9* mutations demonstrated a statistically significant association with tumor site (p = 0.02) and tobacco intake (p = 0.01), suggesting a potential site-specific oncogenic role of *NEK4* and a strong link between *NEK9* mutation and tobacco exposure in OSCC. These associations may aid in understanding the molecular mechanisms of *NEK* gene dysregulation and their relationship with clinicopathological features in oral cancer.

Furthermore, survival analysis revealed distinct trends across the *NEK* gene family. The observed differences in overall survival between *NEK-*mutant and WT OSCC patients likely reflect the context-dependent dual roles of *NEK*s in cancer. Previous studies indicate that *NEK*s can function as either oncogenes or tumor suppressors depending on cellular context and mutation type (Nguyen et al., 2023; Chen et al., 2023; Anuraga et al., 2021). Our findings that *NEK10-*mutant patients exhibited a significantly better overall survival (p < 0.05), whereas mutations in *NEK1*, *NEK2*, *NEK5*, and *NEK9* showed non-significant trends toward improved survival, and mutations in *NEK4* and *NEK8* showed a trend toward worse survival are consistent with a context‐dependent dual role of NEKs in tumorigenesis. *NEK4* generally acts as a tumor suppressor involved in DNA damage response and cell-cycle checkpoint control; thus, its mutation may impair genomic stability and contribute to poorer survival (Park et al., 2016). Similarly, *NEK8* is reported to be overexpressed in\ gastric, colorectal, and breast cancers, where it promotes proliferation and is linked with poor prognosis (Cao et al., 2023; Gao et al., 2022). In contrast, *NEK1*, *NEK2*, *NEK5*, *NEK9*, and *NEK10* often exhibit oncogenic functions, promoting centrosome amplification, mitotic progression, and proliferation; loss-of-function mutations in these genes may weaken their pro-tumorigenic potential, which could explain the better survival of patients with mutant forms (Zhu et al., 2024; Yang et al., 2022). In particular, *NEK10* regulates G_2_/M checkpoint and MAPK signaling, and its impaired function may reduce tumor growth potential, aligning with the improved survival seen in our cohort (Moniz et al., 2011). Collectively, these findings suggest that *NEK* mutations may contribute to heterogeneous clinical outcomes in oral cancer and warrant further validation in larger patient cohorts to clarify their prognostic relevance.

Based on our *in silico* findings, the druggability of *NEK* family mutations remains largely unexplored due to the limited availability of selective *NEK* inhibitors in clinical practice. These mutations are located within or near conserved kinase domains, suggesting a potential impact on catalytic activity or PPIs. However, their presence may modulate cellular signaling pathways, influencing tumor cell sensitivity or resistance to broader classes of kinase inhibitors. Therefore, understanding the functional consequences of these mutations is critical, as they may either serve as future drug targets or act as biomarkers for treatment stratification and therapeutic response. *NEK2* is consistently overexpressed across all types of gastrointestinal cancers, including gastric and pancreatic tumors, and contributes to tumor progression and drug resistance. Currently, T-1101 tosylate, emerging as the most advanced *NEK2* inhibitor, has shown promising preclinical results (Xia et al., 2025). Similarly, *NEK10*, as a potential tumor-promoting factor in lung adenocarcinoma, could serve as a new therapeutic target and warrant further investigation in OSCC (Dutt et al., 2024).

## Conclusion

In conclusion, this study provides the first comprehensive mutational profiling of all 11 *NEK*s in OSCC patients from KPK, revealing novel insights into their mutation spectrum, demographic associations, and potential prognostic implications. Recurrent alterations were most frequent in *NEK1*, *NEK9*, *NEK10*, and *NEK11*, with some localizing to functionally relevant regions. Computational analyses suggest possible effects on protein stability and interactions, though these require experimental confirmation. Observed associations with clinicodemographic factors (naswar use and tumor site) and survival (*NEK10* with improved outcomes) are preliminary and limited by cohort size. Overall, our findings expand the knowledge of *NEK* alterations in OSCC and underscore population-specific patterns, but validation in larger cohorts and functional studies is essential to elucidate mechanistic roles and clinical utility.

Furthermore, this study has certain limitations. The relatively limited sample size posed challenges in establishing robust correlations between molecular findings and clinicopathological parameters. In addition, the study primarily relies on WES and *in silico* analyses without experimental validation. Although *in vitro* or *in vivo* functional assays would provide mechanistic confirmation of the biological impact of *NEK* gene mutations, such analyses were beyond the scope of the present work. Future studies involving larger cohorts from the same population, along with functional validation experiments, are needed to elucidate the mechanistic contributions, prevalence, and clinical relevance of *NEK* mutations in OSCC.

## Data Availability

All the whole exome sequence data is submitted to the NCBI under the project accession number PRJNA1189482 and all the SRA record can be accessed at https://www.ncbi.nlm.nih.gov/bioproject/?term=PRJNA1189482.
