# Supplementary material for: Mutational insights and in silico characterization of NEK family kinases in OSCC patients from the Pakistani population
Source: Front Bioinform. 2026 Feb 4;5:1750649. doi: 10.3389/fbinf.2025.1750649 (PMC12913394; doi:10.3389/fbinf.2025.1750649)
Supplement: Supplementary file 1 [file Table1.docx]

**Mutational Insights and *In-silico* Characterization of *NEK* Family Kinases in Pakistani Population with Oral Squamous Cell Carcinoma**

Fouzia Nawab^1^, Wafa Naeem^1^, Sadia Fatima^1^, Muhammad Uzair Khan^1^, Aamir Mehmood^2^, Sadia Nawab^3,4^, Ishaq Khan^1^, Haseena Nawaz^1^, Hilal Ahmad^1^, Ali Talha Khalil*^5^, Ishtiaq Ahmad Khan^6^, Muhammad Irfan^6^ , Mohammed Alorini^7^, Syed Ali Khurram*^8^, Asif Ali*^7,9,10^


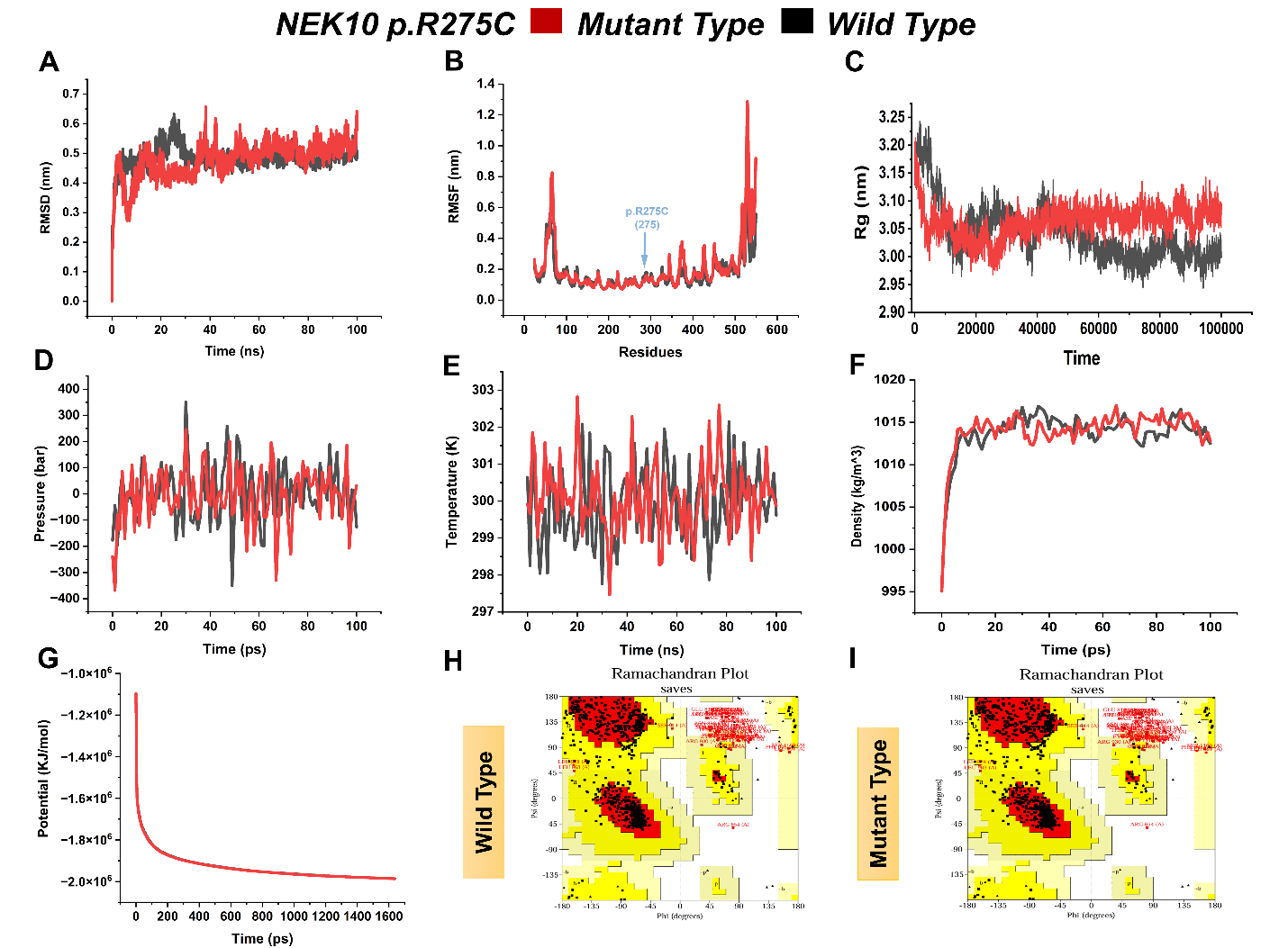


**FIGURE S1.** GROMACS-Based Molecular Dynamics and Structural Validation of Wild-Type and Mutant *NEK10 ^p.R275C^* (A–I)


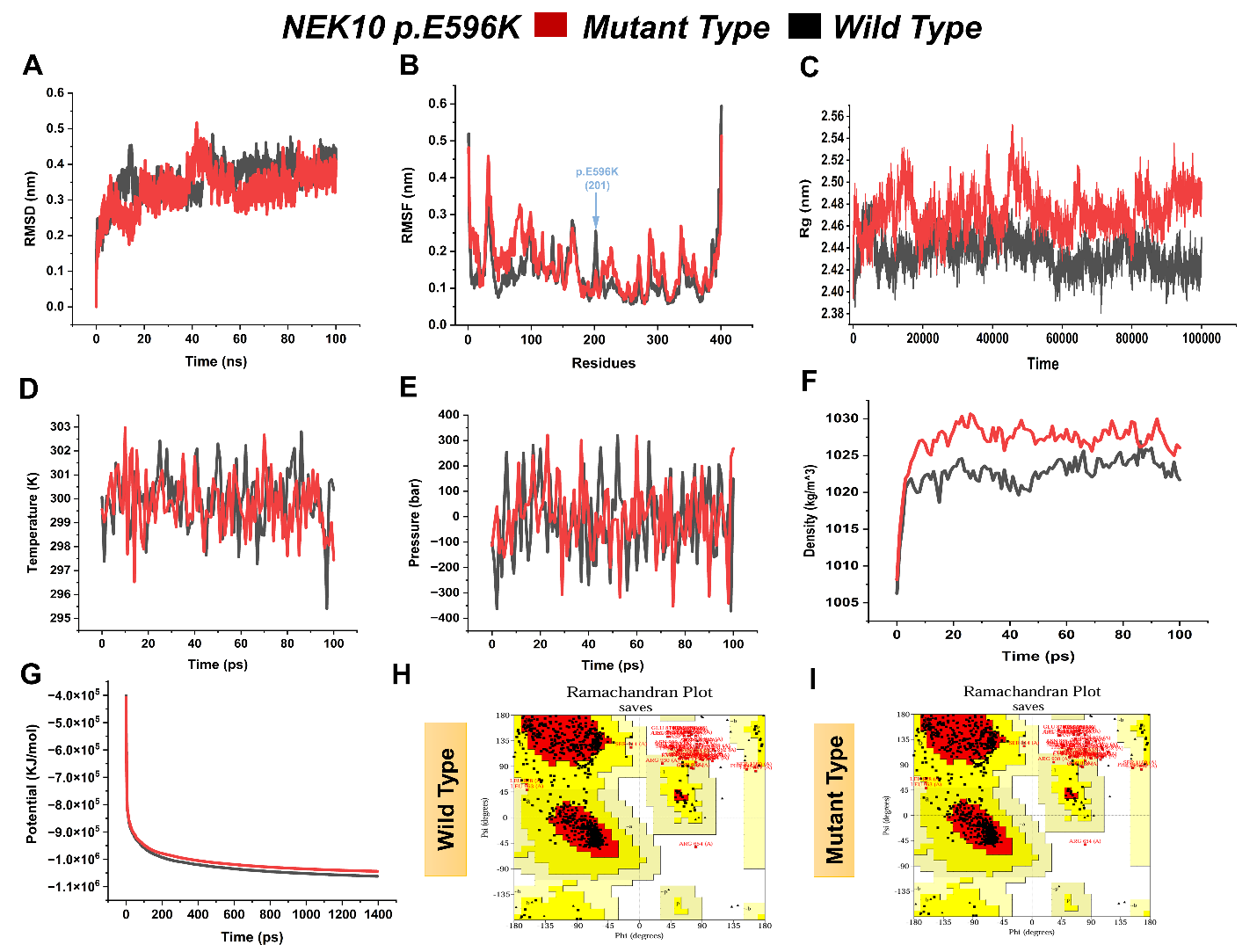


**FIGURE S2.** GROMACS-Based Molecular Dynamics and Structural Validation of Wild-Type and Mutant *NEK10 ^p.E596K^* (A–I)


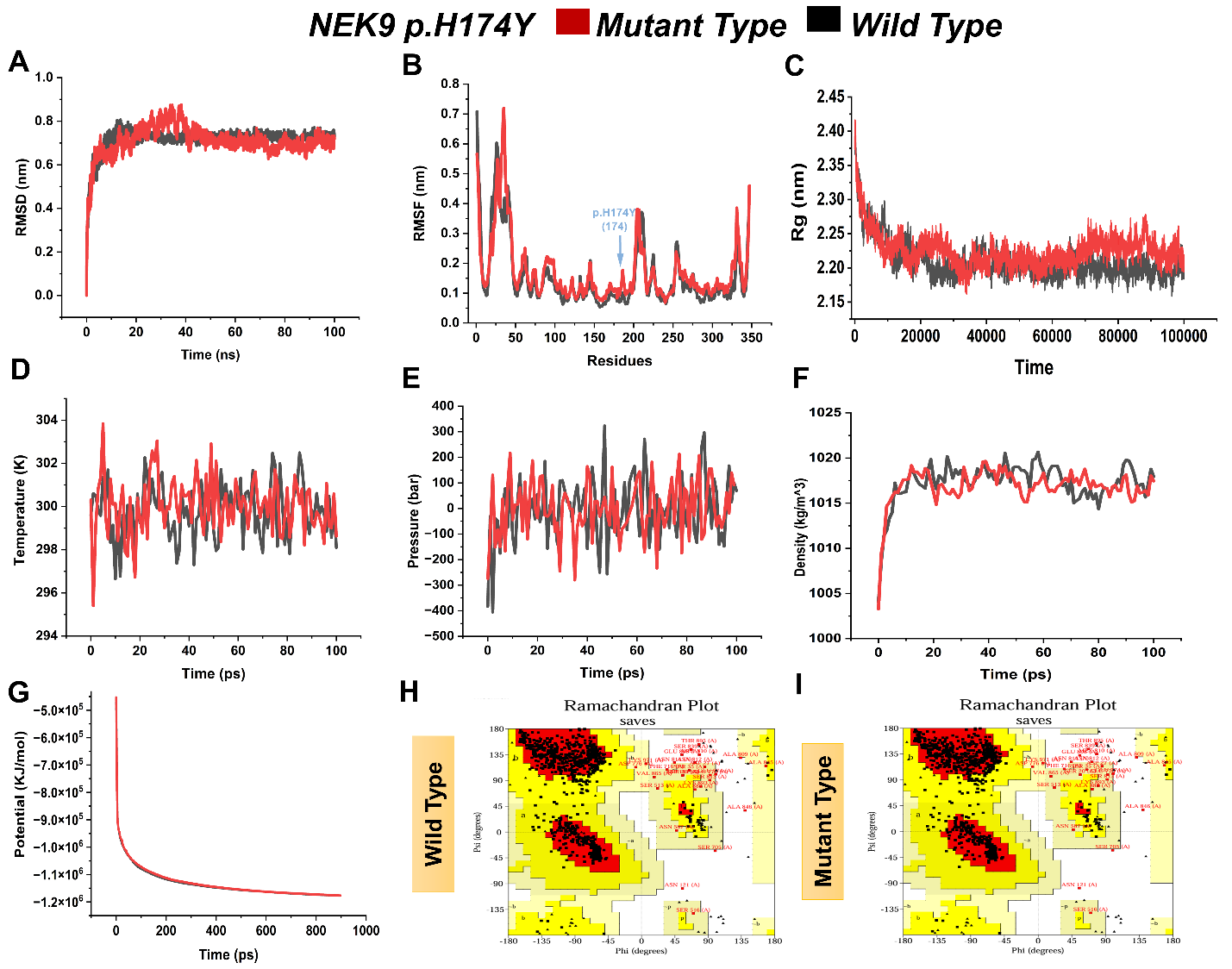


**FIGURE S3.** GROMACS-Based Molecular Dynamics and Structural Validation of Wild-Type and Mutant *NEK9 ^p.H174Y^* (A–I)


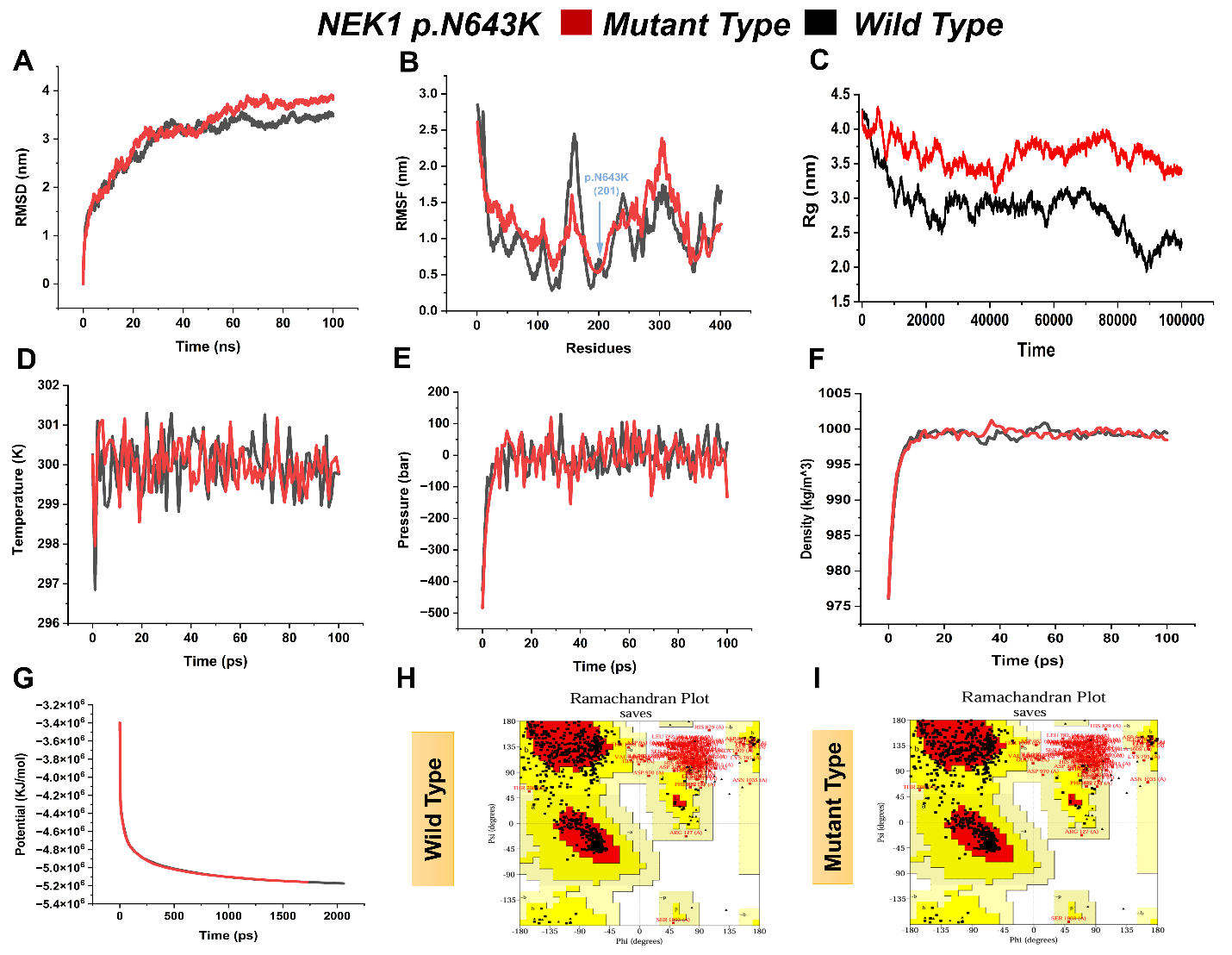


**FIGURE S4.** GROMACS-Based Molecular Dynamics and Structural Validation of Wild-Type and Mutant *NEK1 ^p.N643K^* (A–I)


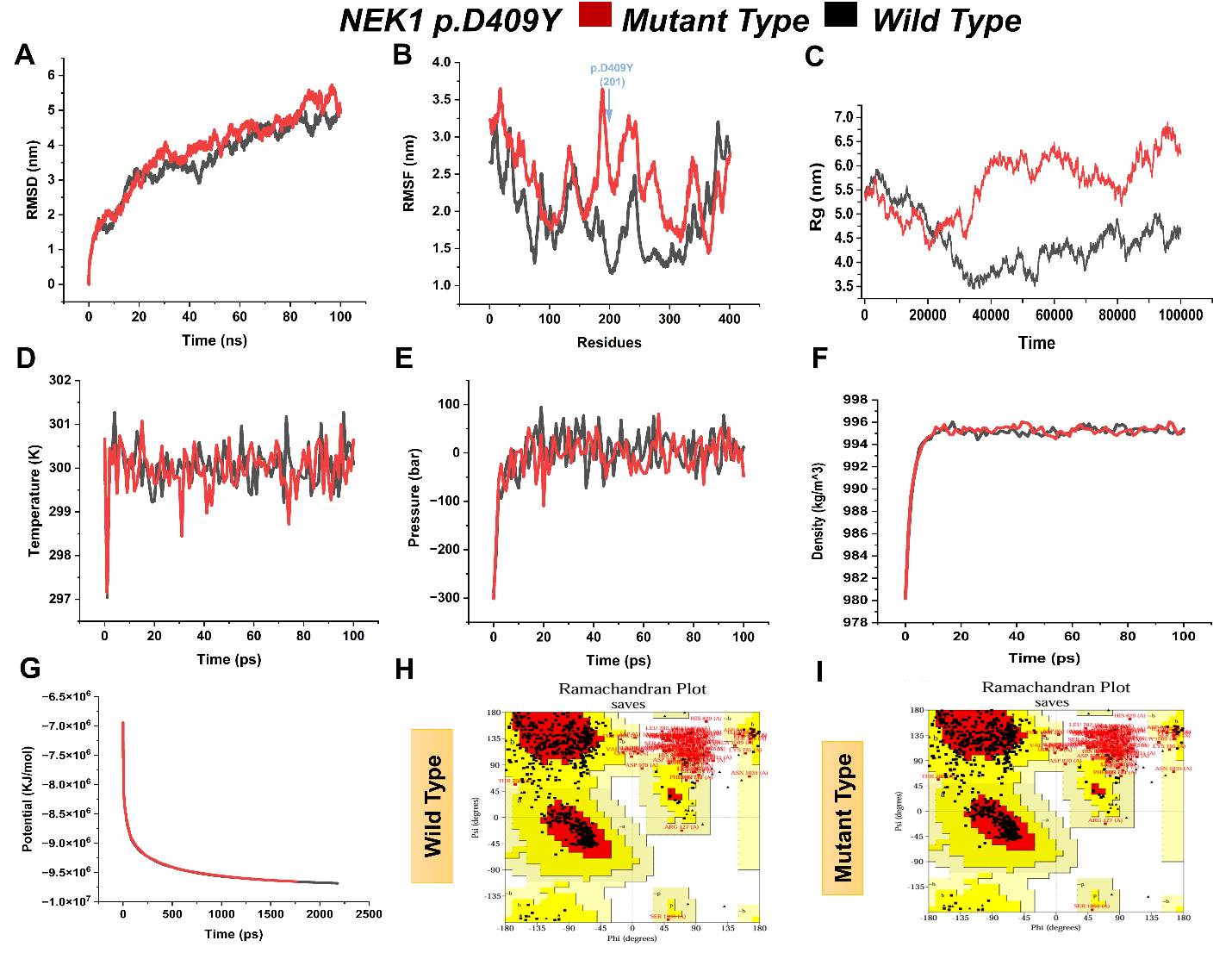


**FIGURE S5.** GROMACS-Based Molecular Dynamics and Structural Validation of Wild-Type and Mutant *NEK1 ^p.D409Y^* (A–I)


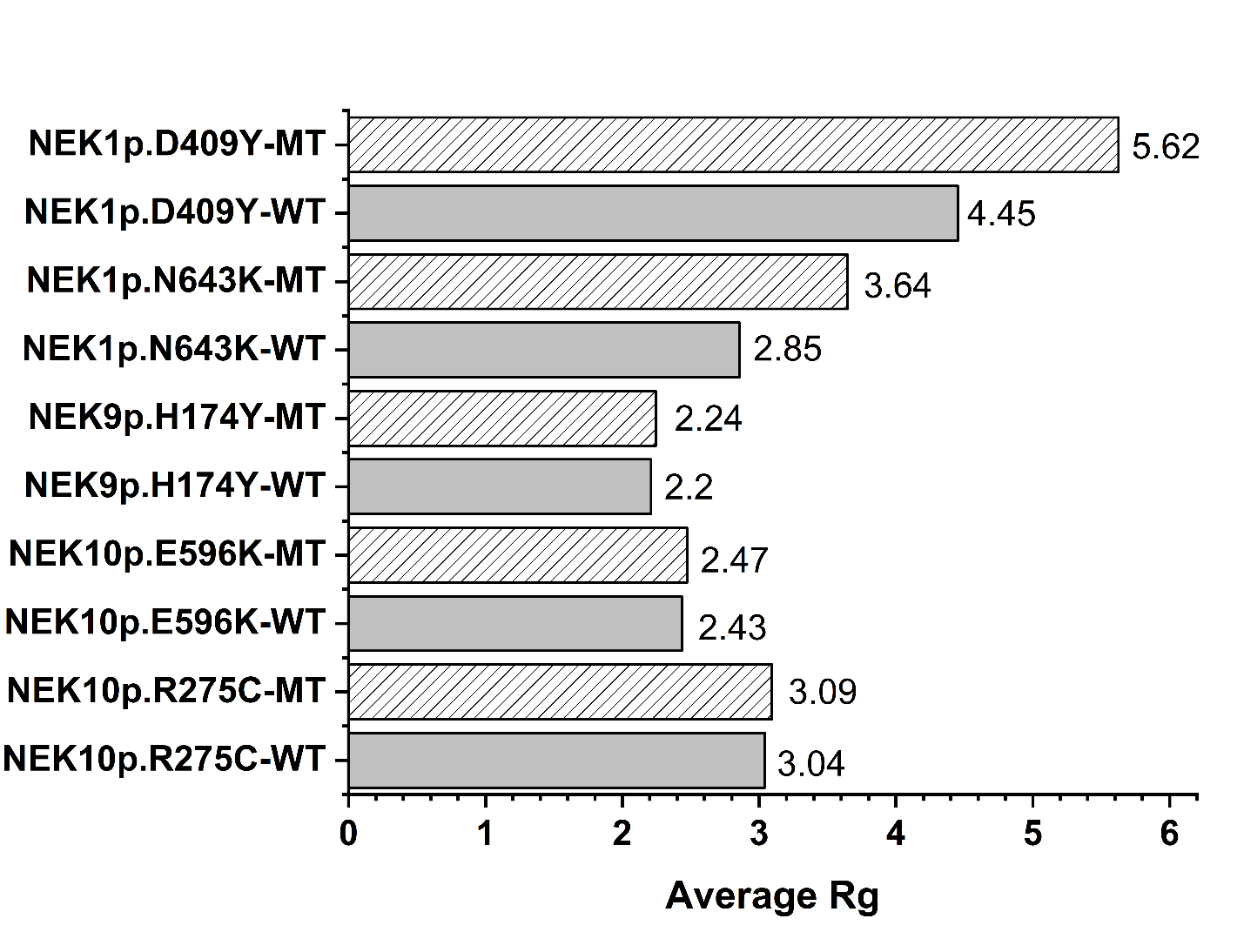


**FIGURE S6.** Average Rg values of Wild-Type and Mutant NEK1, NEK9 and NEK10 Variants


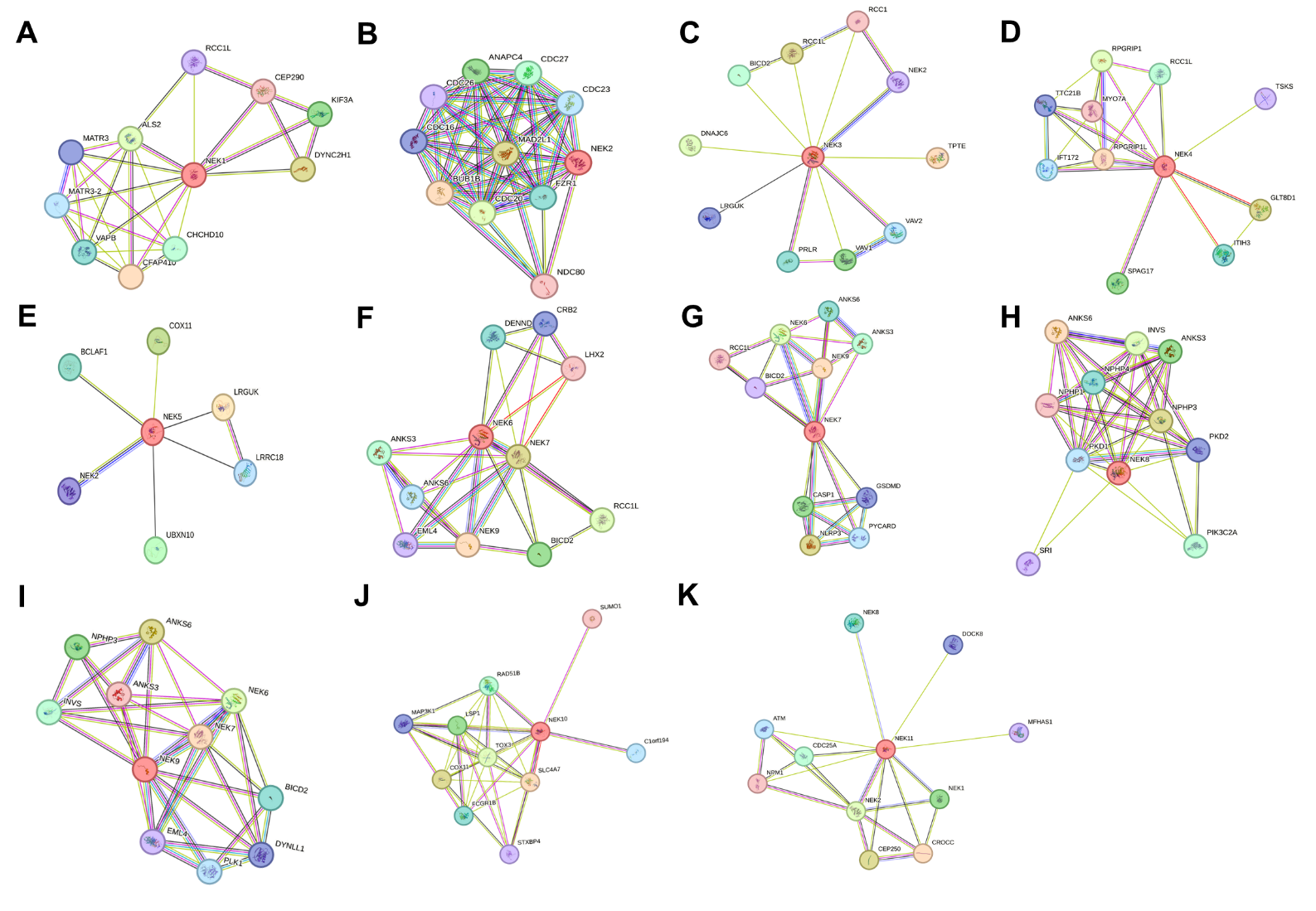


**FIGURE S7.** Protein-protein interaction network of NEK genes as predicted by STRING (A; *NEK1*, B; *NEK2*, C; *NEK3*, D; *NEK4*, E; *NEK5*, F; *NEK6*, G; *NEK7*, H; *NEK8*, I; *NEK9*, J; *NEK10*, K; *NEK11*)

**TABLE S1.** Summary of the Mutational Landscape of NEK Family Genes

| **Patient No** | **Mutation Type** | **Reported** | **Status** | **Exon** | **Nucleotide Change** | **Effect on Protein** | **ClinVar** | **SIFT Pred** | **Polyphen- 2 Pred** | **Mutation Taster Pred** | **Mutation Assessor Pred** | **PROVEAN Pred** | **FATHHM Pred** |
| --- | --- | --- | --- | --- | --- | --- | --- | --- | --- | --- | --- | --- | --- |
|  |  |  |  |  | **NEK1** |  |  |  |  |  |  |  |  |
| 1 | nonsynonymous SNV | dbSNP/ Cosmic | Germline | 19 | c.1585G>A | p.A529T | Benign | T | B | D | L | N | T |
| 1 | nonsynonymous SNV | dbSNP/ Cosmic | Somatic | 25 | c.2308G>A | p.E770K | - | T | B | N | M | N | T |
| 1 | frameshift deletion | Novel | Somatic | 10 | c.808_826del | p.L270Vfs*2 | - | - | - | - | - | - | - |
| 1 | nonsynonymous SNV | - | Somatic | 10 | c.814G>T | p.A272S | - | T | D | D | M | N | T |
| 1 | nonsynonymous SNV | dbSNP | Somatic | 11 | c.884C>T | p.S295L | - | T | P | N | M | N | T |
| 1 | frameshift deletion | Novel | Somatic | 12 | c.1038_1039del | p.K347Efs*13 | - | - | - | - | - | - | - |
| 1 | nonsynonymous SNV | - | Somatic | 12 | c.1064G>A | p.R355K | - | T | B | N | M | N | T |
| 1 | nonsynonymous SNV | dbSNP | Somatic | 15 | c.1298C>G | p.P433R | Benign/Likely benign | D | P | N | L | N | T |
| 1 | nonsynonymous SNV | - | Somatic | 15 | c.1225G>T | **p.D409Y** | - | D | D | D | M | D | T |
| 1 | frameshift deletion | Novel | Somatic | 21 | c.1869delA | p.E624Rfs*19 | - | - | - | - | - | - | - |
| 5 | nonsynonymous SNV | dbSNP | Germline | 22 | c.1949A>G | p.E650G | Benign | T | P | P | M | D | T |
| 2 | nonsynonymous SNV | dbSNP | Somatic | 22 | c.1929T>G | **p.N643K** | Conflicting;Uncertain/ Benign/Likely benign | D | D | D | M | D | D |
| 1 | frameshift deletion | Novel | Somatic | 27 | c.2859delC | p.N953Kfs*48 | - | - | - | - | - | - | - |
| 1 | nonsynonymous SNV | - | Somatic | 27 | c.2858A>T | p.N953I | - | D | B | N | N | N | T |
|  |  |  |  |  | **NEK2** |  |  |  |  |  |  |  |  |
| 1 | nonsynonymous SNV | - | Somatic | 2 | c.244C>A | p.L82M | - | D | D | D | M | N | T |
| 4 | nonsynonymous SNV | dbSNP/ Cosmic | Germline | 7 | c.1061A>G | p.N354S | Benign | T | B | P | L | N | T |
| 1 | frameshift deletion | dbSNP | Somatic | 8 | c.1204_1205del | p.Q403Afs*4 | - | - | - | - | - | - | - |
|  |  |  |  |  | **NEK3** |  |  |  |  |  |  |  |  |
| 1 | nonsynonymous SNV | dbSNP/ Cosmic | Somatic | 14 | c.1307C>T | p.S436L | **-** | **-** | D | N | M | - | - |
| 1 | nonsynonymous SNV | - | Germline | 2 | c.109C>A | p.L37I | - | T | P | D | N | N | T |
| 1 | nonsynonymous SNV | - | Germline | 2 | c.112C>A | p.P38T | - | T | B | D | N | D | T |
|  |  |  |  |  | **NEK4** |  |  |  |  |  |  |  |  |
| 7 | nonsynonymous SNV | dbSNP/ Cosmic | Germline | 4 | c.406C>G | p.P136A | - | D | P | P | L | D | T |
| 4 | nonsynonymous SNV | dbSNP | Somatic | 9 | c.1432T>C | p.F478L | - | T | B | N | N | N | T |
|  |  |  |  |  | **NEK5** |  |  |  |  |  |  |  |  |
| 6 | nonsynonymous SNV | dbSNP | Germline | 10 | c.763A>C | p.K255Q | - | D | D | P | N | N | T |
| 1 | nonsynonymous SNV | - | Somatic | 10 | c.790C>T | p.P264S | - | T | B | N | N | N | T |
| 1 | nonframeshift deletion | Novel | Somatic | 23 | c.2224_2226del | ^p.D742del^ | - | - | - | - | - | - | - |
| 1 | Stopgain | - | Somatic | 18 | c.1601G>A | p.W534X | - | - | - | A | - | - | - |
| 1 | nonsynonymous SNV | dbSNP | Somatic | 18 | c.1639G>A | p.A547T | - | T | B | N | N | N | T |
| 1 | nonsynonymous SNV | - | Somatic | 18 | c.1621T>C | p.W541R | - | T | B | N | N | N | T |
|  |  |  |  |  | **NEK6** |  |  |  |  |  |  |  |  |
| 1 | nonsynonymous SNV | dbSNP/ Cosmic | Somatic | 10 | c.925C>G | p.H309D | - | T | B | N | N | N | T |
|  |  |  |  |  | **NEK7** |  |  |  |  |  |  |  |  |
| 1 | Stopgain | - | Somatic | 5 | c.299C>A | p.S100X | - | - | - | A | - | - | - |
|  |  |  |  |  | **NEK8** |  |  |  |  |  |  |  |  |
| 1 | nonsynonymous SNV | - | Somatic | 6 | c.864C>A | p.S288R | - | T | B | N | M | N | T |
| 1 | nonsynonymous SNV | dbSNP | Somatic | 5 | c.805G>A | p.V269M | - | T | B | N | L | N | T |
|  |  |  |  |  | **NEK9** |  |  |  |  |  |  |  |  |
| 23 | nonsynonymous SNV | dbSNP | Germline | 11 | c.1286G>A | p.R429H | Benign | T | B | P | N | N | D |
| 1 | nonsynonymous SNV | - | Somatic | 4 | c.520C>T | **p.H174Y** | - | D | D | D | M | D | T |
|  |  |  |  |  | **NEK10** |  |  |  |  |  |  |  |  |
| 1 | nonsynonymous SNV | dbSNP | Somatic | 10 | c.920T>C | p.V307A | - | T | B | D | - | N | T |
| 1 | nonsynonymous SNV | dbSNP | Somatic | 11 | c.1030A>G | p.I344V | - | T | B | N | - | N | T |
| 1 | nonsynonymous SNV | dbSNP/ Cosmic | Somatic | 13 | c.823C>T | **p.R275C** | - | D | D | D | L | D | T |
| 15 | nonsynonymous SNV | dbSNP/ Cosmic | Germline | 19 | c.1538T>C | p.L513S | Benign | T | B | P | L | N | T |
| 1 | nonsynonymous SNV | - | Somatic | 21 | c.1786G>A | **p.E596K** | - | D | D | D | L | D | T |
| 1 | nonsynonymous SNV | dbSNP | Somatic | 25 | c.2110C>T | p.R704C | - | D | - | N | - | N | T |
| 1 | nonsynonymous SNV | - | Somatic | 25 | c.2111G>T | p.R704L | - | D | - | N | - | N | T |
|  |  |  |  |  | **NEK11** |  |  |  |  |  |  |  |  |
| 1 | nonsynonymous SNV | dbSNP | Somatic | 6 | c.253G>A | p.A85T | - | T | B | D | N | N | T |
| 31 | nonsynonymous SNV | dbSNP | Germline | 10 | c.1040A>T | p.E347V | - | T | D | P | M | D | T |
| 3 | nonsynonymous SNV | dbSNP | Somatic | 12 | c.1262T>C | p.V421A | - | T | B | P | N | N | T |
| 3 | nonsynonymous SNV | dbSNP | Somatic | 14 | c.1415A>G | p.K472R | - | T | B | N | - | N | T |
| 3 | nonsynonymous SNV | dbSNP | Somatic | 14 | c.1403C>T | p.A468V | - | T | B | N | - | N | T |

**Legends: “SIFT”: D:Deleterious; T: Tolerated; “PolyPhen-2”: D: Probably damaging; P: Possibly damaging; B Benign; “Mutation Taster”: A: Disease causing automatic; D: Disease causing; N: Polymorphism; P: Polymorphism automatic; “Mutation Assessor”: L: Low; M: Medium; N: Neutral; “PROVEAN”: N: Neutral; D: Deleterious ;“FATHMM”: D: Deleterious ; T: Tolerated**

**TABLE S2.** SAAFEC-SEQ Predictions of Mutation-Induced Protein Stability Changes

| Position | Wild type Residue | Mutant type residue | ddG Unit | Predicted Effect |
| --- | --- | --- | --- | --- |
| NEK1 |  |  |  |  |
| p.E770K | E | K | -0.73 | Destabilizing |
| p.A529T | A | T | -1.21 | Destabilizing |
| p.A272S | A | S | -1.07 | Destabilizing |
| p.S295L | S | L | -0.38 | Destabilizing |
| p.R355K | R | K | -0.81 | Destabilizing |
| p.P433R | P | R | -1.36 | Destabilizing |
| p.D409Y | D | Y | -0.71 | Destabilizing |
| p.E650G | E | G | -1.36 | Destabilizing |
| p.N643K | N | K | -0.70 | Destabilizing |
| p.N953I | N | I | -0.15 | Destabilizing |
| NEK2 |  |  |  |  |
| p.L82M | L | M | -1.28 | Destabilizing |
| p.N354S | N | S | -0.37 | Destabilizing |
| NEK3 |  |  |  |  |
| p.S436L | S | L | -0.18 | Destabilizing |
| p.L37I | L | I | -1.39 | Destabilizing |
| p.P38T | P | T | -0.90 | Destabilizing |
| NEK4 |  |  |  |  |
| p.P136A | P | A | -1.04 | Destabilizing |
| p.F478L | F | L | -1.42 | Destabilizing |
| NEK5 |  |  |  |  |
| p.K255Q | K | Q | -0.40 | Destabilizing |
| p.P264S | P | S | -0.45 | Destabilizing |
| p.W534X | W | X | - | - |
| p.A547T | A | T | -1.12 | Destabilizing |
| p.W541R | W | R | -0.66 | Destabilizing |
| NEK6 |  |  |  |  |
| p.H309D | H | D | -0.11 | Destabilizing |
| NEK7 |  |  |  |  |
| p.S100X | S | X | - | - |
| NEK8 |  |  |  |  |
| p.S288R | S | R | -0.05 | Destabilizing |
| p.V269M | V | M | -0.74 | Destabilizing |
| NEK9 |  |  |  |  |
| p.R429H | R | H | -1.05 | Destabilizing |
| p.H174Y | H | Y | -0.95 | Destabilizing |
| NEK10 |  |  |  |  |
| p.V307A | V | A | -0.95 | Destabilizing |
| p.I344V | I | V | -0.47 | Destabilizing |
| p.R275C | R | C | -0.62 | Destabilizing |
| p.L513S | L | S | -0.89 | Destabilizing |
| p.E596K | E | K | -0.86 | Destabilizing |
| p.R704C | R | C | -0.48 | Destabilizing |
| p.R704L | R | L | -0.43 | Destabilizing |
| NEK11 |  |  |  |  |
| p.A85T | A | T | -0.74 | Destabilizing |
| p.E347V | E | V | -0.19 | Destabilizing |
| p.V421A | V | A | -0.40 | Destabilizing |
| p.K472R | K | R | -0.26 | Destabilizing |
| p.A468V | A | V | -0.41 | Destabilizing |

**TABLE S3.** List of predicted Interaction Sites Mutations by ISPRED-SEQ

| S.No | Mutation | Prediction | Probability |
| --- | --- | --- | --- |
| NEK1 |  |  |  |
| 1 | p.A529T | N | 0.39 |
| 2 | p.E770K | N | 0.37 |
| 3 | p.A272S | N | 0.47 |
| 4 | p.S295L | N | 0.13 |
| 5 | p.R355K | **IS** | 0.51 |
| 6 | p.P433R | **IS** | 0.54 |
| 7 | p.D409Y | **IS** | 0.59 |
| 8 | p.E650G | N | 0.44 |
| 9 | p.N643K | **IS** | 0.67 |
| 10 | p.N953I | N | 0.4 |
| NEK2 |  |  |  |
| 1 | p.L82M | N | 0.1 |
| 2 | p.N354S | **IS** | 0.71 |
| NEK3 |  |  |  |
| 1 | p.S436L | N | 0.44 |
| 2 | p.L37I | N | 0.39 |
| 3 | p.P38T | N | 0.49 |
| NEK4 |  |  |  |
| 1 | p.P136A | N | 0.34 |
| 2 | p.F478L | **IS** | 0.65 |
| NEK5 |  |  |  |
| 1 | p.K255Q | N | 0.34 |
| 2 | p.P264S | N | 0.45 |
| 3 | p.W534X | **IS** | 0.74 |
| 4 | p.A547T | **IS** | 0.61 |
| 5 | p.W541R | **IS** | 0.67 |
| NEK6 |  |  |  |
| 1 | p.H309D | **IS** | 0.64 |
| NEK7 |  |  |  |
| 1 | p.S100X | **IS** | 0.69 |
| NEK8 |  |  |  |
| 1 | p.S288R | N | 0.14 |
| 2 | p.V269M | **IS** | 0.58 |
| NEK9 |  |  |  |
| 1 | p.R429H | N | 0.23 |
| 2 | p.H174Y | **IS** | 0.52 |
| NEK10 |  |  |  |
| 1 | p.V307A | **IS** | 0.55 |
| 2 | p.I344V | **IS** | 0.64 |
| 3 | p.R275C | **IS** | 0.6 |
| 4 | p.L513S | N | 0.27 |
| 5 | p.E596K | **IS** | 0.52 |
| 6 | p.R704C | N | 0.5 |
| 7 | p.R704L | N | 0.5 |
| NEK11 |  |  |  |
| 1 | p.A85T | N | 0.46 |
| 2 | p.E347V | **IS** | 0.52 |
| 3 | p.V421A | **IS** | 0.66 |
| 4 | p.K472R | N | 0.23 |
| 5 | p.A468V | N | 0.16 |

***Legends:*** *IS; Interacting sites, N; non-interacting site*

**TABLE S4.** Evolutionary Conservation Profiles of NEK Genes Predicted by ConSurf

| **S.No** | **Mutations** | **Conservation Scores (1-9)** | **Predicted Findings** | **ConSurf Predictions** |
| --- | --- | --- | --- | --- |
| 1. | NEK1p.R355K | 4 | Moderately variable status and exposed residue | 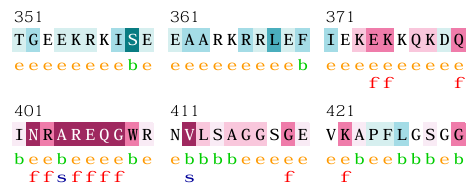 |
| 2. | NEK1p.P433R | 1 | Highly variable status and exposed residue | 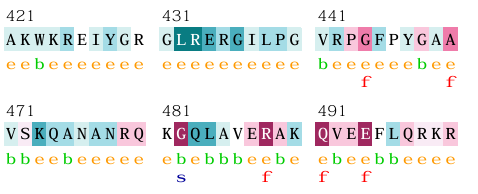 |
| 3 | NEK1p.D409Y | 6 | Moderately conserved status and buried residue | 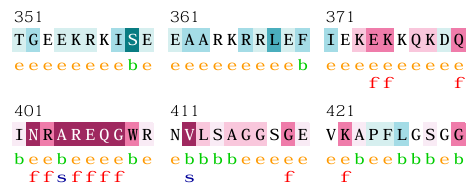 |
| 4 | NEK1p.N643K | 8 | Highly conserved status and exposed residue | 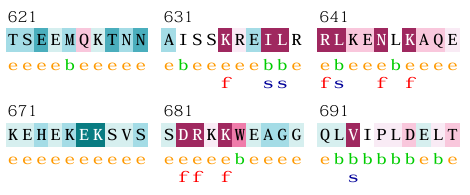 |
| 5 | NEK2p.N354S | 4 | Moderately variable status and exposed residue | 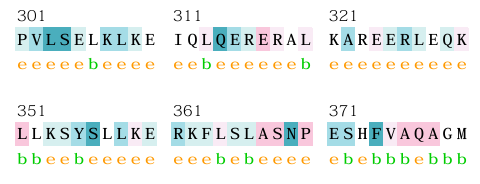 |
| 6 | NEK4 p.F478L | 6 | Moderately conserved status and exposed residue | 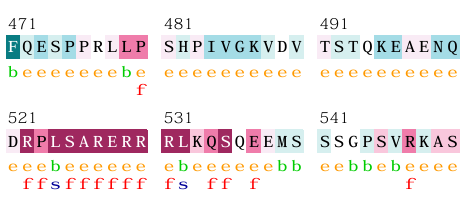 |
| 7 | NEK5p.A547T | 5 | Average status and buried residue | 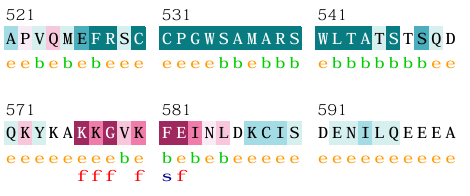 |
| 8 | NEK5p.W541R | 6 | Moderately conserved status and exposed residue | 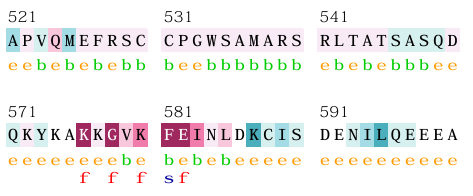 |
| 9 | NEK6p.H309D | 2 | Highly variable status and exposed residue | 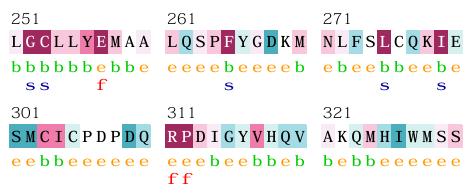 |
| 10 | NEK8p.V269M | 5 | Average conservation score and buried residue | 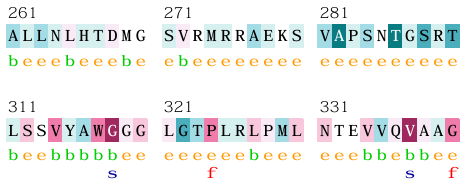 |
| 11 | NEK9 p.H174Y | 9 | Highly conserved status and buried/ residue | 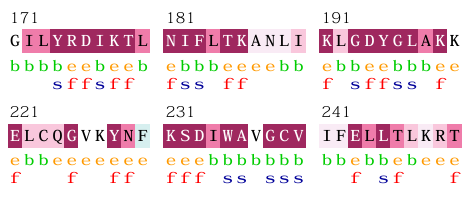 |
| 12 | NEK10p.V307A | 4 | Moderately variable status and buried residue | 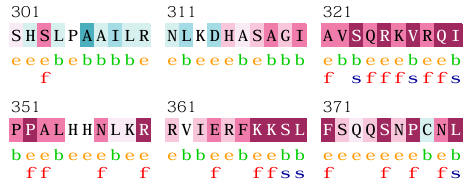 |
| 13 | NEK10 p.I344V | 7 | Moderately conserved status and buried residue | 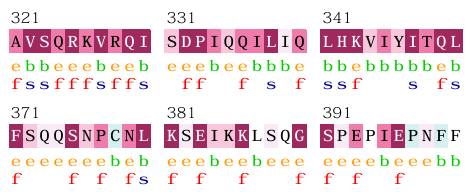 |
| 14 | NEK10p.R275C | 8 | Highly conserved status and exposed/functional residue | 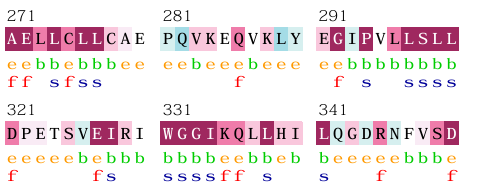 |
| 15 | NEK10p.E596K | 8 | Highly conserved status and exposed/functional residue | 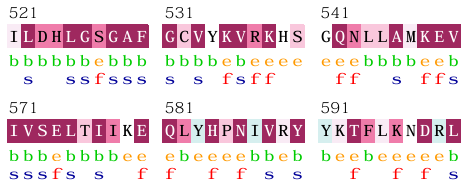 |
| 16 | NEK11p.E347V | 5 | Average conservation score and buried residue | 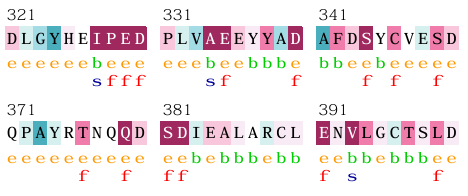 |

**Table S5A.** Association of NEK Genes with Clinico-pathological and Demographic Parameters

| **Characteristics** |  | **NEK1** | | | **p-value** | **NEK2** | | | **p-value** | **NEK4** | | | **p-value** |
| --- | --- | --- | --- | --- | --- | --- | --- | --- | --- | --- | --- | --- | --- |
|  |  | **Present** | **Absent** | **Total** |  | **Present** | **Absent** | **Total** |  | **Present** | **Absent** | **Total** |  |
| **Age** | **≤56** | 7 | 6 | 13 | **0.32** | 3 | 10 | 13 | 0.4 | 5 | 8 | 13 | **0.4** |
|  | **>56** | 7 | 11 | 18 |  | 3 | 15 | 18 |  | 5 | 13 | 18 |  |
| **Gender** | **Male** | 11 | 11 | 22 | **0.33** | 3 | 19 | 22 | **0.3** | 7 | 15 | 22 | **0.6** |
|  | **Female** | 3 | 6 | 9 |  | 3 | 6 | 9 |  | 3 | 6 | 9 |  |
| **Tumor Grade** | **Well** | 5 | 10 | 15 | **0.1** | 4 | 11 | 15 | **0.3** | 4 | 11 | 15 | **0.3** |
|  | **Moderate** | 9 | 17 | 16 |  | 2 | 14 | 16 |  | 6 | 10 | 16 |  |
| **Tumor Site** | **Lip** | 2 | 3 | 5 | **0.9** | 2 | 3 | 5 | **0.2** | 1 | 4 | 5 | **0.02** |
|  | **Buccal Mucosa** | 3 | 3 | 6 |  | 1 | 5 | 6 |  | 5 | 1 | 6 |  |
|  | **Tongue** | 5 | 6 | 11 |  | 3 | 8 | 11 |  | 3 | 8 | 11 |  |
|  | **Others** | 4 | 5 | 9 |  | 0 | 9 | 9 |  | 1 | 8 | 9 |  |
| **Tobacco Intake** | **Naswar User** | 9 | 9 | 18 | **0.4** | 2 | 16 | 18 | **0.1** | 6 | 12 | 18 | **0.8** |
|  | **Smoking** | 0 | 2 | 2 |  | 0 | 2 | 2 |  | 1 | 1 | 2 |  |
|  | **Non tobacco users** | 5 | 6 | 11 |  | 4 | 7 | 11 |  | 3 | 8 | 11 |  |
| **Family History** | **Present** | 7 | 6 | 13 | **0.3** | 2 | 11 | 13 | **1.00** | 4 | 9 | 13 | **1.00** |
|  | **Absent** | 7 | 11 | 18 |  | 4 | 14 | 18 |  | 6 | 12 | 18 |  |
| **Dental Problem History** | **Yes** | 6 | 4 | 10 | **0.2** | 1 | 9 | 10 | **0.6** | 2 | 8 | 10 | **0.2** |
|  | **No** | 8 | 13 | 21 |  | 5 | 16 | 21 |  | 8 | 13 | 21 |  |

**Table S5B.** Association of NEK Genes with Clinico-pathological and Demographic Parameters (continued)

| **Characteristics** |  | **NEK5** | | | **p-value** | **NEK9** | | | **p-value** | **NEK10** | | | **p-value** |
| --- | --- | --- | --- | --- | --- | --- | --- | --- | --- | --- | --- | --- | --- |
|  |  | **Present** | **Absent** | **Total** |  | **Present** | **Absent** | **Total** |  | **Present** | **Absent** | **Total** |  |
| **Age** | **≤56** | 4 | 9 | 13 | **0.44** | 9 | 4 | 13 | **0.6** | 5 | 8 | 13 | **0.1** |
|  | **>56** | 4 | 14 | 18 |  | 14 | 4 | 18 |  | 12 | 6 | 18 |  |
| **Gender** | **Male** | 7 | 15 | 22 | **0.23** | 16 | 6 | 22 | **1.00** | 14 | 8 | 22 | **0.1** |
|  | **Female** | 1 | 8 | 9 |  | 7 | 2 | 9 |  | 3 | 6 | 9 |  |
| **Tumor Grade** | **Well** | 5 | 10 | 15 | **0.3** | 10 | 5 | 15 | **0.4** | 9 | 6 | 15 | **0.4** |
|  | **Moderate** | 3 | 13 | 16 |  | 13 | 3 | 16 |  | 8 | 8 | 16 |  |
| **Tumor Site** | **Lip** | 1 | 4 | 5 | **0.9** | 4 | 1 | 5 |  | 2 | 3 | 5 | **0.3** |
|  | **Buccal Mucosa** | 2 | 4 | 6 |  | 5 | 1 | 6 | **0.7** | 2 | 4 | 6 |  |
|  | **Tongue** | 3 | 8 | 11 |  | 7 | 4 | 11 |  | 8 | 3 | 11 |  |
|  | **Others** | 2 | 7 | 9 |  | 7 | 2 | 9 |  | 5 | 4 | 9 |  |
| **Tobacco intake** | **Naswar User** | 5 | 13 | 18 | **0.61** | 16 | 2 | 18 | **0.01** | 10 | 8 | 18 | **0.9** |
|  | **Smoking** | 1 | 1 | 2 |  | 0 | 2 | 2 |  | 1 | 1 | 2 |  |
|  | **Non tobacco users** | 2 | 9 | 11 |  | 7 | 4 | 11 |  | 6 | 5 | 11 |  |
| **Family History** | **Present** | 4 | 9 | 13 | **0.6** | 10 | 3 | 13 | **1.000** | 8 | 5 | 13 | **0.3** |
|  | **Absent** | 4 | 14 | 18 |  | 13 | 5 | 18 |  | 9 | 9 | 18 |  |
| **Dental Problem History** | **Yes** | 2 | 8 | 10 | **0.48** | 8 | 2 | 10 | **1.000** | 5 | 5 | 10 | **0.5** |
|  | **No** | 6 | 15 | 21 |  | 15 | 6 | 21 |  | 12 | 9 | 21 |  |

**Table S6.** Comparison of NEK genes mutation frequencies between the present cohort and TCGA-HNSCC

| **Gene** | **Mutated Samples present Cohort (n=31)** | **Frequency (%)** | **Mutated Samples TCGA-HNSCC (n= 515)** | **Frequency %** |
| --- | --- | --- | --- | --- |
| NEK1 | 13 | 41.9% | 7 | 1.4% |
| NEK2 | 6 | 19.4% | 3 | 0.6% |
| NEK3 | 2 | 6.5% | 1 | 0.2% |
| NEK4 | 11 | 35.5% | 2 | 0.4% |
| NEK5 | 9 | 29.0% | 13 | 2.5% |
| NEK6 | 1 | 3.2% | 1 | 0.2% |
| NEK7 | 1 | 3.2% | 1 | 0.2% |
| NEK8 | 2 | 6.5% | 6 | 1.2% |
| NEK9 | 23 | 74.2% | 4 | 0.8% |
| NEK10 | 17 | 54.8% | 6 | 1.2% |
| NEK11 | 31 | 100% | 5 | 1% |
